# Investigating Engineered Ribonucleoprotein Particles to Improve Oral RNAi Delivery in Crop Insect Pests

**DOI:** 10.3389/fphys.2017.00256

**Published:** 2017-04-28

**Authors:** François-Xavier Gillet, Rayssa A. Garcia, Leonardo L. P. Macedo, Erika V. S. Albuquerque, Maria C. M. Silva, Maria F. Grossi-de-Sa

**Affiliations:** ^1^Embrapa Genetic Resources and BiotechnologyBrasília, Brazil; ^2^Department of Cellular Biology, Brasilia Federal University (UnB)Brasília, Brazil; ^3^Graduate Program in Genomics and Biotechnology, Catholic University of BrasiliaBrasilia, Brazil

**Keywords:** insect pest, oral dsRNA delivery, gut nucleases, PTD-DRBD, dsRNA protection, RNA interference (RNAi)

## Abstract

Genetically modified (GM) crops producing double-stranded RNAs (dsRNAs) are being investigated largely as an RNA interference (RNAi)-based resistance strategy against crop insect pests. However, limitations of this strategy include the sensitivity of dsRNA to insect gut nucleases and its poor insect cell membrane penetration. Working with the insect pest cotton boll weevil (*Anthonomus grandis*), we showed that the chimeric protein PTD-DRBD (peptide transduction domain—dsRNA binding domain) combined with dsRNA forms a ribonucleoprotein particle (RNP) that improves the effectiveness of the RNAi mechanism in the insect. The RNP slows down nuclease activity, probably by masking the dsRNA. Furthermore, PTD-mediated internalization in insect gut cells is achieved within minutes after plasma membrane contact, limiting the exposure time of the RNPs to gut nucleases. Therefore, the RNP provides an approximately 2-fold increase in the efficiency of insect gene silencing upon oral delivery when compared to naked dsRNA. Taken together, these data demonstrate the role of engineered RNPs in improving dsRNA stability and cellular entry, representing a path toward the design of enhanced RNAi strategies in GM plants against crop insect pests.

## Introduction

The Cotton boll weevil (*Anthonomus grandis*) is an economically important crop insect pest that attacks cotton fields, particularly in South America (de Lima et al., 2013). The larvae live and feed off the cotton flowers and buds, causing serious damage to cotton yields. This endophytic habit is incompatible with chemical pesticide treatments, and no simple or efficient alternatives have been discovered to date (Ribeiro et al., 2010; Neves et al., 2014).

Developing resistant cotton plants is considered the best alternative for preventing boll weevil attacks. For this purpose, the use of the RNA interference (RNAi) has been applied to generate genetically modified (GM) plants resistant to different pests (Baum et al., 2007; Mao et al., 2011; Zhang et al., 2015). This RNAi-based approach relies on double-stranded RNAs (dsRNAs), which are recognized as signaling molecules for the gene silencing machinery in almost all eukaryotic organisms (Meister and Tushi, 2004; Meister, 2013). When an insect feeds on GM plants, the ingestion of long-length dsRNAs causes the silencing of essential genes in a sequence specific manner, triggering death or abnormal progeny development (Price and Gatehouse, 2008; Burand and Hunter, 2013; Katoch et al., 2013; Kim et al., 2015). Therefore, the generation of GM plants producing dsRNA could be a sustainable, efficient and specific resistance strategy against crop insect pests.

While the microinjection of dsRNA into the insect body cavity is often reported to be efficient for gene silencing, its oral delivery in *A. grandis* and other insects is still a challenge (Baum et al., 2007; Bellés, 2010; Katoch and Thakur, 2012; Shukla et al., 2016). Indeed, nucleases in the insect gut lumen degrade dsRNA, considerably diminishing its gene silencing efficiency (Arimatsu et al., 2007a,b; Katoch and Thakur, 2012; Wynant et al., 2014; Joga et al., 2016). Another major obstacle to the passage of dsRNA molecules from the gut lumen into cells is their poor gut epithelium permeability. The plasma membrane of the gut epithelial cells functions as a barrier, separating the cytoplasm from the extracellular environment. The negative charge of dsRNA prevents the passive membrane transport of the molecule into the cell. In some insects, dsRNA internalization depends on the SID-1 dsRNA transporter and/or endosomal trafficking at the interface between the lumen and the gut cells (Price and Gatehouse, 2008; Burand and Hunter, 2013; Katoch et al., 2013). The length of dsRNA is an additional important penetration parameter as short dsRNAs (24 nts) are not taken up by midgut cells (Bolognesi et al., 2012; Li et al., 2015; Joga et al., 2016). Therefore, insect gut nucleases and dsRNA uptake are crucial obstacles that are currently being examined to improve oral RNAi strategies against crop insect pests.

The intracellular delivery of dsRNA using carrier systems is currently being investigated in the biomedical and pharmaceutical fields (Prokop et al., 2014). These carriers comprise molecules with different biochemical properties that can improve dsRNA stability, endosomal lysis and/or membrane penetration. Cell-penetrating peptides (CPPs) are of particular interest because they can improve dsRNA stability and/or cell entry (Milletti, 2012; Prokop et al., 2014). Interestingly, CPPs have been investigated for the delivery of bioactive molecules into insect cells (Cermenati et al., 2011; Chen et al., 2012; Zhou et al., 2015). Given that CPPs can be fused with proteins of interest, it is tempting to speculate that these bioactive molecules can be produced in plants (Hughes et al., 2012; Kwon and Daniell, 2016). In the CPP family, the arginine-rich Tat peptide has been specifically studied and genetically engineered to improve its efficiency. The peptide transduction domain (PTD) is an enhanced version of the Tat peptide. This domain includes the lipid fusogenic properties of the hemagglutinin (HA) peptide, which destabilizes the vesicle membrane after endocytosis, dispersing the molecule into the cytoplasm (Wadia et al., 2004; Erazo-Oliveras et al., 2012). Fusing PTD with the dsRNA binding domain (DRBD) of human protein kinase R (PKR) (Eguchi et al., 2009) improves small interfering RNA (siRNA) delivery. When combined, PTD and DRBD allow the formation of a ribonucleoprotein particle (RNP) capable of being internalized by the cell via endocytosis, escaping the endosome to deliver dsRNA into the cytoplasm and therefore triggering the silencing of the targeted gene (Wadia et al., 2004; Eguchi et al., 2009). Remarkably, no cytotoxicity and minimal off-target transcriptional changes have been observed in human cells treated with micromolar concentrations of PTD-DRBD combined with siRNA (Eguchi et al., 2009). While the use of engineered RNPs has been reported to improve RNAi technologies in various animal cells, whether they could improve RNAi technology for crop insect pest management remains an open question.

Here, we show that the complex PTD-DRBD:dsRNA enhances gene silencing after oral dsRNA administration in *A. grandis*. The binding of PTD-DRBD to long dsRNAs results in the formation of RNPs that protect the dsRNA against gut nucleases. Moreover, gut cells internalize the RNPs on a remarkably short time scale. This parameter contributes to dsRNA stability, considering that the less time the dsRNA stays in the gut lumen, the fewer the chances of it being exposed to nucleases. The feasibility of producing PTD-DRBD and its usefulness as a dsRNA carrier system that functions via RNP formation *in planta* will be discussed.

## Results

### Complex formation of long-length dsRNA with PTD-DRBD

**Figure 1:**
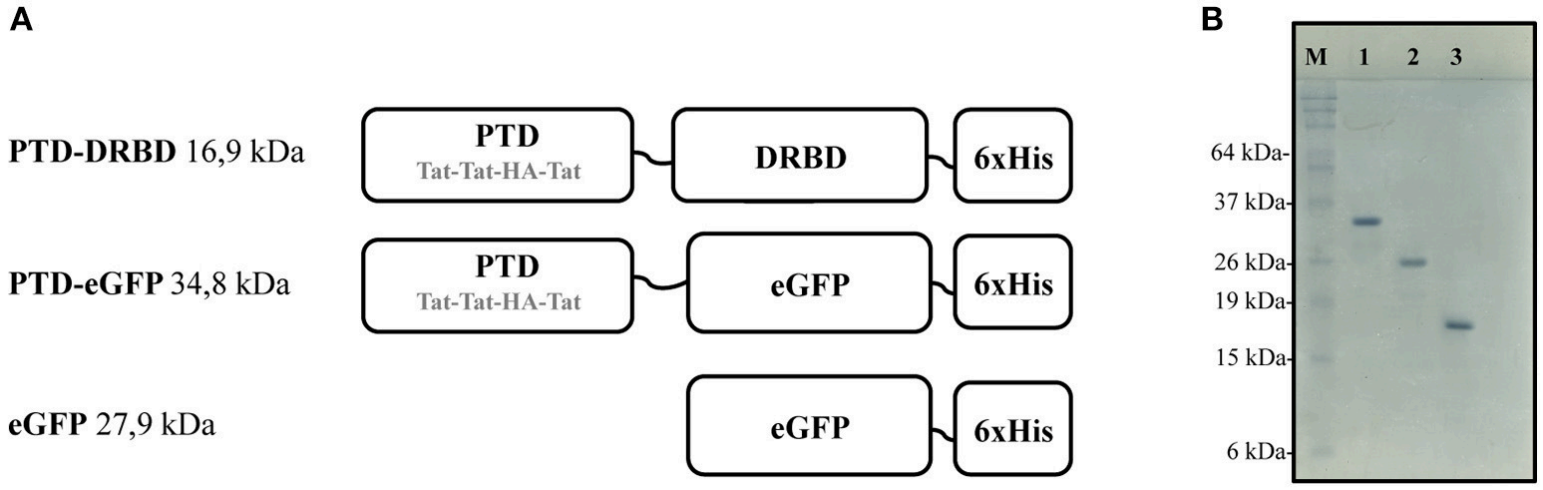
**Recombinant proteins constructions and respective gel migration patterns. (A)** Representation of the PTD domain composed by three repeated arginine rich Tat peptides and the fusogenic HA peptide. The DRBD or eGFP domain is fused to PTD in the N-terminal region. Short linker regions are indicated by black lines. **(A)** C-terminal 6xHis tag (6xHis) is used for affinity purification. **(B)** SDS-PAGE analysis showing PTD-eGFP (1), eGFP (2), and PTD-DRBD (3) after purification. PTD, protein transduction domain; DRBD, double strand RNA-binding domain; eGFP, enhanced green fluorescent protein.

**Figure 2:**
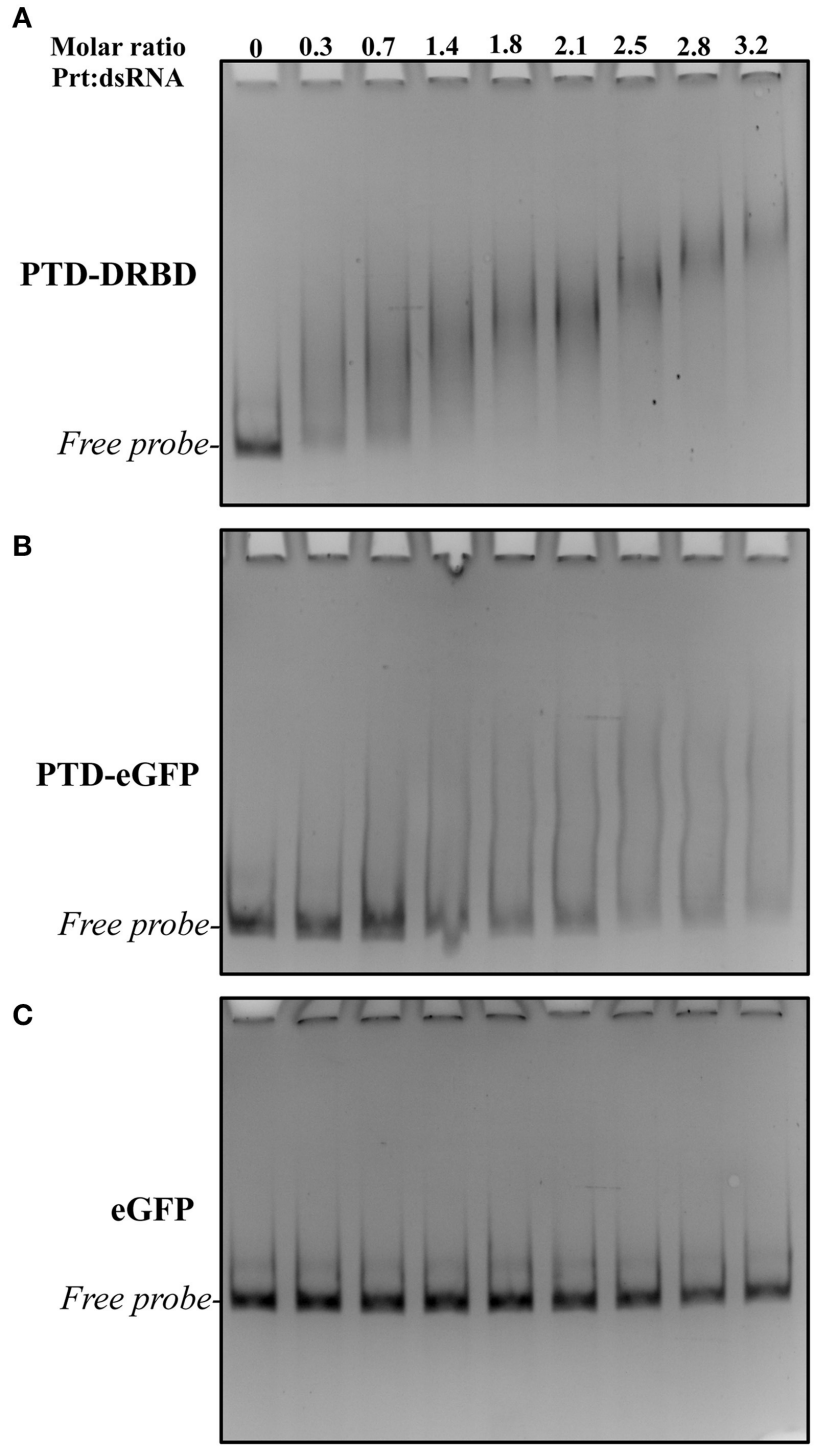
**EMSA analysis of long dsRNA and different recombinant proteins**. The assay was performed at various recProtein:dsRNA molar ratios and concentrations. The dsRNA was incubated with different concentrations of recProteins PTD-DRBD **(A)**, PTD-eGFP **(B)**, and eGFP **(C)** and their interaction was evaluated by electrophoresis followed by ethidium bromide staining. Each concentration of recProteins ranged from 25 to 225 nM with a constant dsRNA concentration of 70 nM.

Gene silencing in insects has been reported to be effective when using long-length dsRNAs (60–200 nts) (Bolognesi et al., 2012), in contrast to the short-length siRNA (20–30 nts) commonly used in molecular therapies. The binding of PTD-DRBD with dsRNA has only been evaluated for small RNAs (24 nts) (Eguchi et al., 2009; Geoghegan et al., 2012). Firstly, we report to what extend PTD-DRBD binds to long dsRNA by performing an electrophoretic mobility shift assay (EMSA). The assay was carried out using recombinant PTD-DRBD purified from *Escherichia coli* (Figure 1) and a 185-bp RNA duplex. Titration of the PTD-DRBD:dsRNA complex at nanomolar concentrations resulted in the formation of mobility shift bands visible more as a smear than as specific band shifts (Figure 2A). The protein-dsRNA complex mobility decreased as the PTD-DRBD concentration increased. As expected, a similar result was obtained when using a distinct sequence of a 150-bp RNA duplex, indicating that PTD-DRBD binding is not sequence specific (Figure S1A). We also observed that the complex was retained well at a low PTD-DRBD:dsRNA molar ratio (approximately 3:1) (Figure S1B). This result suggests that PTD-DRBD might bind to more than one dsRNA molecule, resulting in the formation of multimeric complexes or aggregates. It has been reported that the positive charges carried by the Tat peptide in PTD lead to oligonucleotide binding (Futaki, 2006; Geoghegan et al., 2012; Milletti, 2012). To determine the effect of PTD on dsRNA binding, we performed EMSA assays with the recombinant fusion protein PTD-eGFP and with only eGFP, which is a non-dsRNA binding protein (Figures 1, 2B,C). The assays were conducted with identical protein-dsRNA molar ratios and concentrations. PTD-eGFP binding to dsRNA was detected as a slight but continuous smear starting from the free probe. No binding activity was detected for eGFP. These data indicate that while DRBD is mainly responsible for dsRNA binding under these conditions, PTD may also contribute to complex formation. This was also visible at higher concentration of dsRNA and recombinant proteins in the presence of ethidium bromide (EtBr) (Figure S2). However, PTD-DRBD still bound to dsRNA more efficiently than PTD-eGFP in these conditions. Overall, these results indicate that PTD-DRBD, but also PTD alone with a lower affinity, binds to long-length dsRNA in a dose-dependent and non-sequence-specific manner.

### Stability of the PTD-DRBD:dsRNA complex

**Figure 3:**
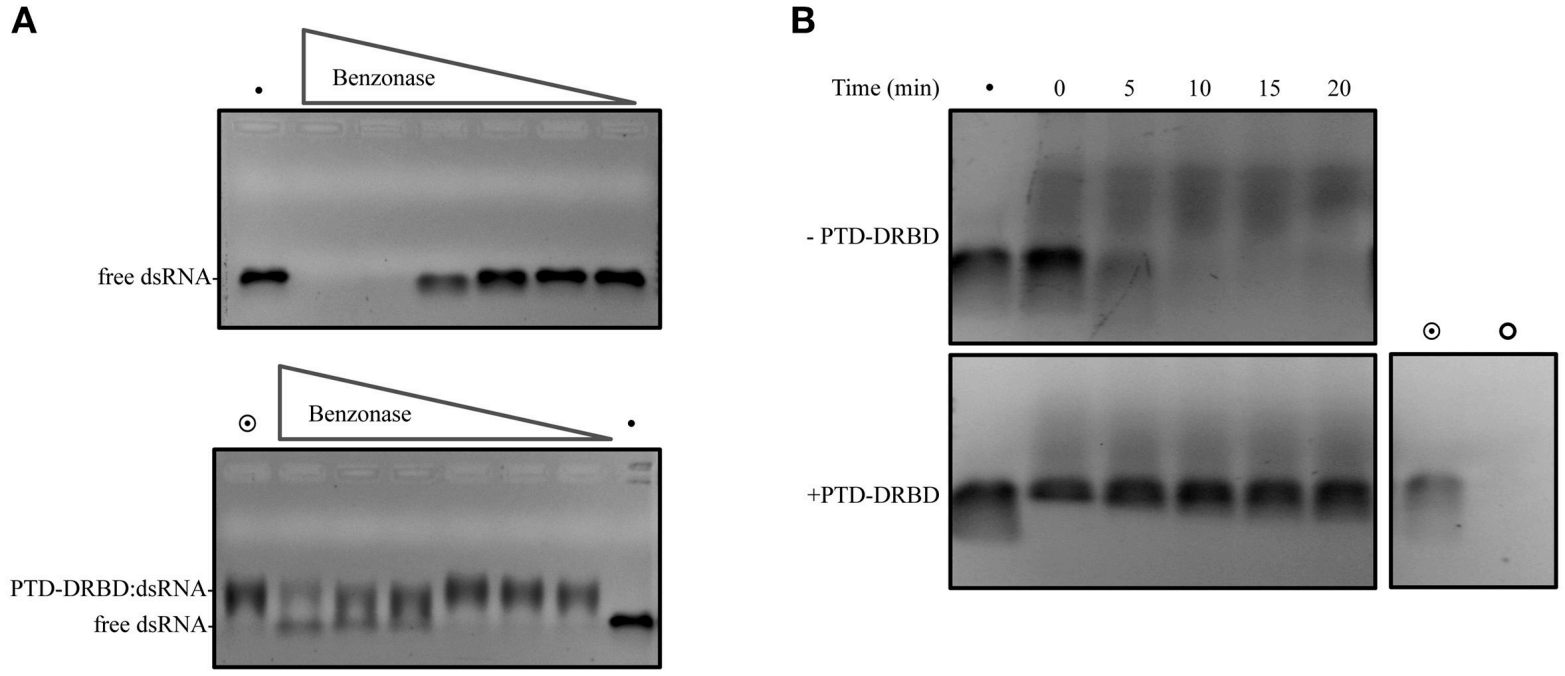
**dsRNA cleavage assay mediated by nuclease**. The complex PTD-DRBD:dsRNA was assembled, with PTD-DRBD at 3.5 μM and dsRNA at 0.4 μM. **(A)** The complex was incubated with benzonase with a dilution starting from one unit until 1:40. Degradation of the dsRNA was analyzed without PTD-DRBD (upper panel) and with PTD-DRBD (lower panel). **(B)** DsRNA was incubated without (upper panel) or with PTD-DRBD (lower panel) before to be incubated with *A. grandis* midgut homogenate at pH5.5. Different aliquots were removed over time and analyzed by electrophoresis. The symbols “•”, “
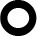
” and “
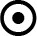
” indicate respectively to naked dsRNA, PTD-DRBD alone and PTD-DRBD: dsRNA.

The strategy of oral dsRNA delivery involves the exposure of dsRNA to the gut lumen environment. Here, one crucial obstacle is the presence of gut nucleases. We speculated that the interaction of PTD-DRBD with dsRNA might offer protection against nuclease activity by covering the dsRNA. It has been reported that insects secrete gut nucleases that belong to the sugar non-specific metal finger family of endonucleases (Wynant et al., 2014). We also identified the heterologous genes coding for this nuclease family in *A. grandis* (Garcia et al., unpublished), which share strong similarities with the nuclease in *Serratia marcescens*, also referred to by the trade name benzonase (Rangarajan and Shankar, 2001; Wynant et al., 2014). We first established a simple assay to examine whether PTD-DRBD could protect dsRNA against nuclease activity. This assay involved incubating the RNPs with serial dilutions of benzonase. Then, dsRNA stability was analyzed by electrophoresis. Notably, protein binding to nucleic acids results in less ethidium bromide (EtBr) fluorescence signal emission, limiting this assay only to qualitative analysis (Zaitseva et al., 1999). At higher benzonase concentrations, dsRNA was detected only when PTD-DRBD was added (Figure 3A). We next challenged the stability of the RNPs via incubation with *A. grandis* midgut homogenate nucleases. The insect midgut was dissected to extract the homogenate (Figure S3). Using color-fixed pH indicator strips, we observed that the pH of the midgut homogenate is comprised between 5 and 6 (Figure S4). The RNPs were incubated with the midgut homogenate buffered at pH 5.5, and dsRNA degradation was followed over time (Figure 3B). In this assay samples were pretreated with SDS before electrophoresis to allow PTD-DRBD dissociation from dsRNA. Notably, SDS interfered a bit with dsRNA electrophoresis. Without PTD-DRBD, a complete degradation of naked dsRNA occurred after 5 min at pH 5.5 (Figure 3B—upper panel). Despite a slight degradation was detectable with PTD-DRBD, the signal corresponding to dsRNA was for mostly preserved. Although the assay has been performed *in vitro* using a diluted midgut homogenate, our data suggest that PTD-DRBD slows down but does not completely counteract *A. grandis* gut nuclease activity.

### Intracellular delivery of PTD-eGFP and PTD-DRBD:dsRNA

**Figure 4:**
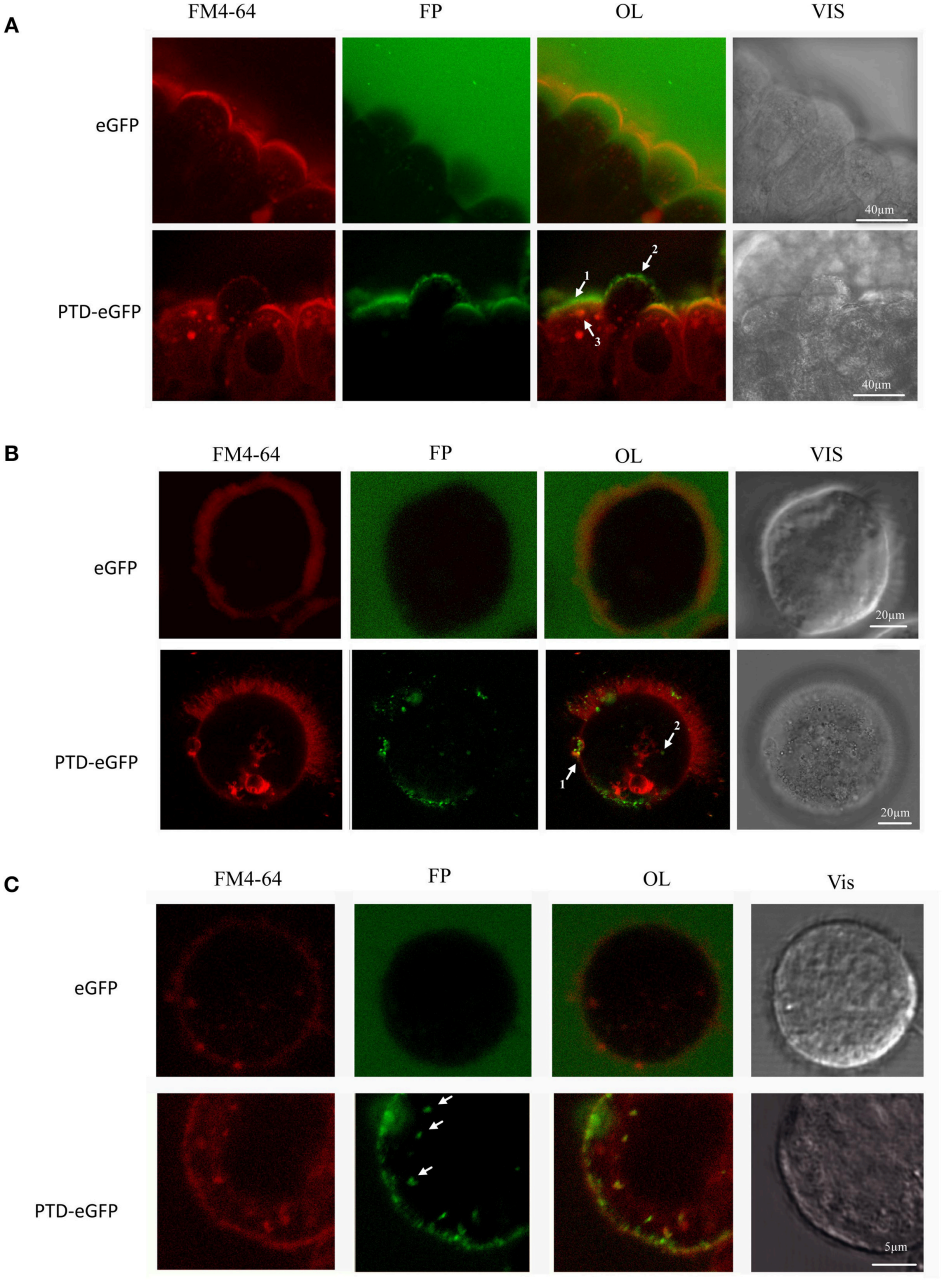
**Live-cell imaging of *A. grandis* midgut and sf21 cells treated with PTD-eGFP**. *A. grandis* midgut was pre-treated with endocytic fluorescent marker FM4-64 (red fluorescence) before incubation with 0.14 μM PTD-eGFP or eGFP. For the experiment with eGFP, the detection sensitivity was increased to allow eGFP observation in the media. **(A)** Whole *A. grandis* midgut treated with PTD-eGFP. In OL arrows indicate the layer of PTD-eGFP on the surface of the plasma membrane (1), co-localization of PTD-eGFP with FM4-64 forming a punctuate pattern on the surface of the plasma membrane (2) and in an endovesicle (3). **(B)** *A. grandis* ciliated cell treated with PTD-eGFP fusion protein. In OL, arrows indicate the formation of a 10 μm cup-shaped plasma membrane modification (1) and co-localization of PTD-eGFP with an endovesicle (2). **(C)** Sf21 cell treated with PTD-eGFP. Arrows show small endovesicles in co-localization with PTD-eGFP. OL, overlay; FP, fluorescent protein (green); Vis, visible light.

Thereafter, we evaluated the ability of PTD-eGFP to penetrate *A. grandis* gut cells using confocal microscopy. Working with a dissected midgut, we observed that eGFP alone was diffuse in the media, while PTD-eGFP formed a layer and a bright and punctuated pattern at the surface of the plasma membrane (Figure 4A). Inside the cell, PTD-eGFP was co-localized with endovesicles, which reached a size of approximately 5 μm, as revealed by the general endocytosis marker FM4-64. Having established that PTD-DRBD slows down but does not completely suppress the nuclease activity of the *A. grandis* midgut homogenate, it remained important to consider time as a crucial component of the mechanism. Hence, we examined the time scale necessary for PTD to penetrate insect cells. To better observe PTD-eGFP internalization, we generated a suspension of *A. grandis* gut cells and added PTD-eGFP directly onto the cells deposited on the microscope blade. It is notable that a delay of 1–2 min was necessary to capture the event and adjust the microscope's parameters. PTD-eGFP clustered at the membrane of the gut cells before starting the acquisition, meaning that the contact after the inoculation occurs in less than 2 min (Movie 1). PTD-eGFP was also detected in co-localization with FM4-64 within endovesicles (Movie 1 and Figure 4B). Moreover, long cup-shaped extensions of the membrane surface could be observed over a distance of 10 μm (Figure 4). We also reported PTD-eGFP internalization on a similar time scale in *Spodoptera frugiperda* 21 (Sf21) cells, which are derived from ovarian tissue. Remarkably, in these cells, the endovesicles reached a size below 1 μm (Figure 4C). We also observed large plasma membrane extensions in contact with PTD-eGFP (Movie 2). Overall, these results indicate that PTD enables internalization by insect cells in a manner independent of their identity and, importantly, on a time scale similar to that necessary for PTD-DRBD to still be protecting dsRNA against the *A. grandis* midgut homogenate nucleases.

**Figure 5:**
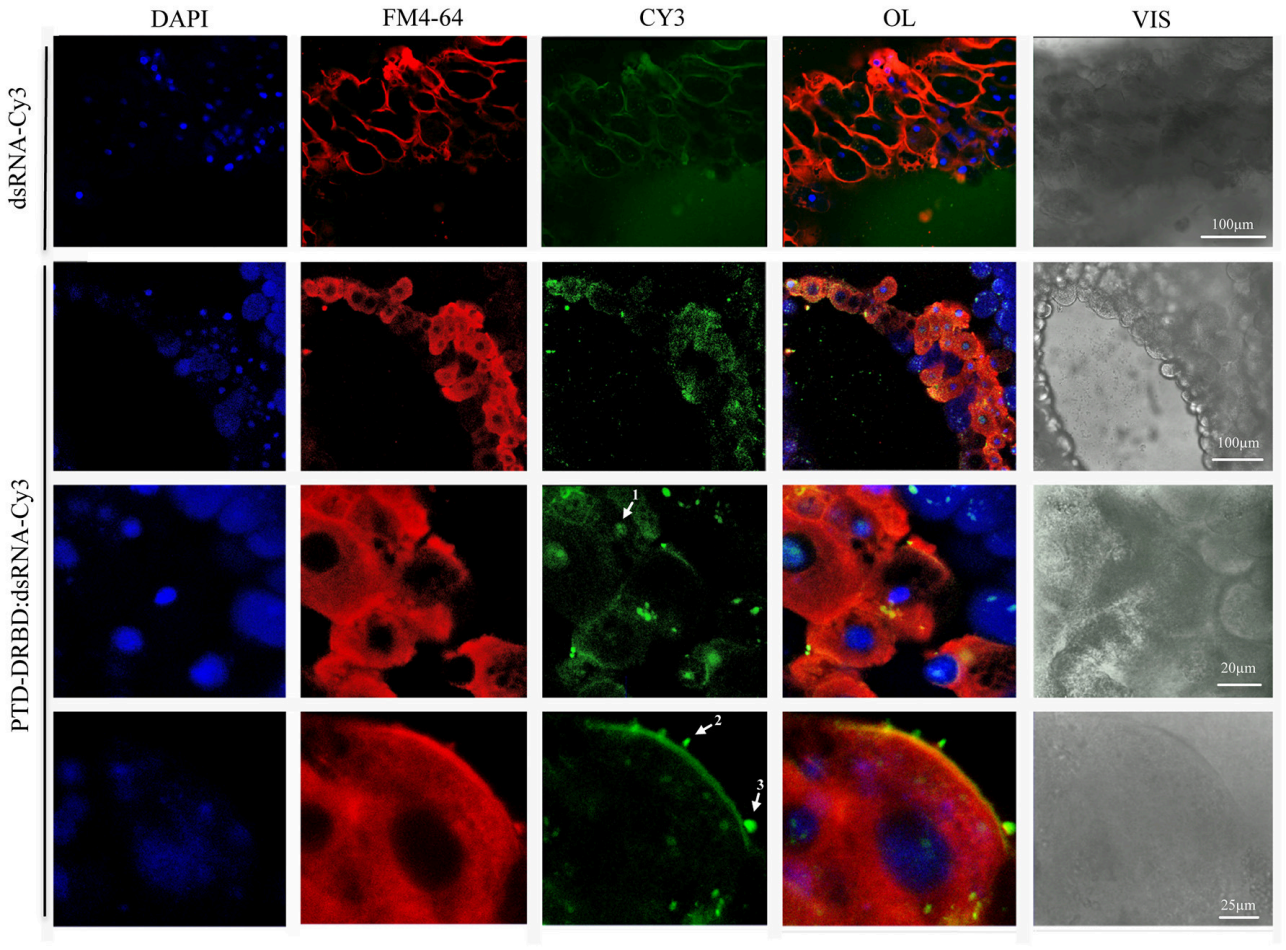
**Internalization of PTD-DRBD:dsRNA by *A. grandis* midgut cells**. *A. grandis* midgut was pre-treated with the fluorescent markers FM4-64 and DAPI before incubation with PTD-DRBD (3 μM) in complex with dsRNA labeled with Cy3 (0.4 μM). The arrows show the detection of dsRNA in the nucleus (1), co-localized with plasma membrane extensions (2) and endovesicles (3). For the experiment with dsRNA-Cy3 alone, the detection sensitivity was increased to allow its observation in the media. DAPI, nuclei blue staining; FM4-64 membrane marker (red); OL, overlay; Cy3, fluorescent protein (green); Vis-DIC, visible light by differential interference contrast (DIC).

To evaluate the internalization of the RNP in gut cells, *A. grandis* midgut was incubated with PTD-DRBD complexed with Cy3-labeled dsRNA (Figure 5). As a control, the assay was reproduced with dsRNA-Cy3 alone. In general, we observed a wide distribution of labeled PTD-DRBD:dsRNA particles concentrated on the cell membrane surface, creating a layer ranging in size from 0.5 to 2 μm. The particles were associated with plasma membrane extensions and within endovesicles, similar to what was observed previously with PTD-eGFP. Moreover, the Cy3-labeled dsRNA was detected in the cytoplasm as a diffuse signal and in nuclei in co-localization with DAPI. PTD has similarities with nuclear localization signals (NLSs), which probably explains the observation of the RNPs in nuclei (Shen et al., 2007; Chugh et al., 2010). Collectively, these data revealed that PTD-DRBD allows internalization of dsRNA by *A. grandis* gut cells via an endocytic pathway and later releases the dsRNA into the cytoplasm.

### Oral delivery of the PTD-DRBD:dsRNA complex to insect gut cells

**Figure 6:**
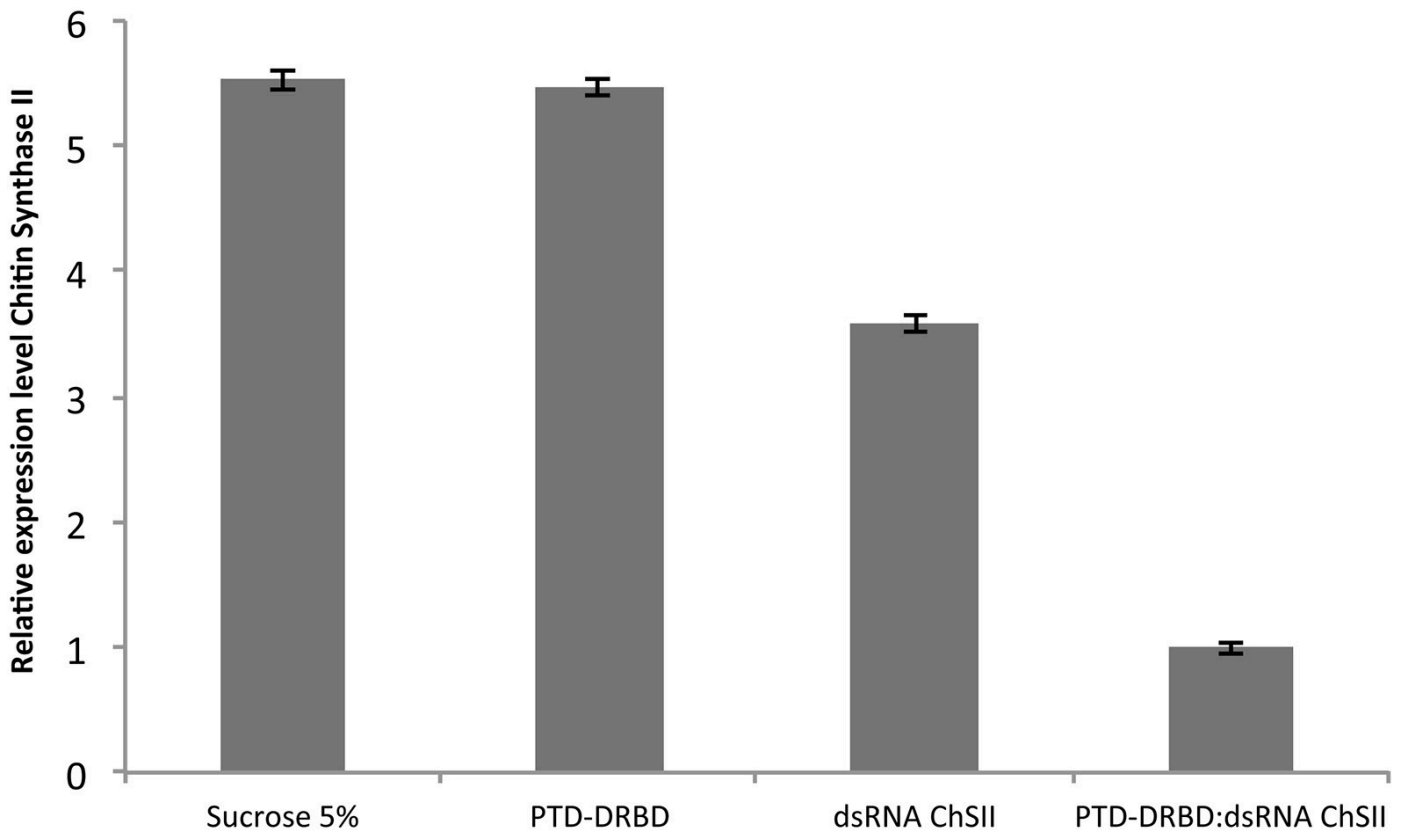
**Relative expression analysis of the *Ag-ChSII* by qRT-PCR in the *A. grandis* midgut 2 days after PTD-DRBD:dsRNA ingestion**. The RNP was formed with 7 μM PTD-DRBD and 0.6 μM dsRNA before oral administration in *A. grandis*. All qRT-PCR experiments were performed with two biological replicates and three technical repetitions. Statistical analyses were performed using Tukey's test with a 0.05% significance level for comparison among treatments. β*-Actin* was used as the reference gene (Firmino et al., 2013).

We next examined whether PTD-DRBD could affect gene silencing by dsRNA oral delivery to *A. grandis*. Some insect genes from the chitin synthase family are essential for insect physiology and development, including the protection of the gut from mechanical damage and invasive parasites, the neutralization of ingested toxins and the facilitation of digestion (Merzendorfer and Zimoch, 2003; Merzendorfer, 2006; Hegedus et al., 2009; Toprak et al., 2016). Therefore, the midgut chitin synthase has been reported to be an ideal insect growth regulatory target for RNAi (Zhang et al., 2010; Jin et al., 2015). To evaluate the influence of PTD-DRBD on insect gene silencing in *A. grandis* by dsRNA oral delivery, we used a 185-nt dsRNA targeting the *A. grandis* chitin synthase II gene (*Ag-ChSII*). *A. grandis* was fed with a saccharose solution containing the PTD-DRBD:dsRNA complex or each compound separately. The *Ag-ChSII* expression level was evaluated by RT-qPCR 2 days after dsRNA ingestion (Figure 6). The expression of *Ag-ChSII* was not significantly different between the insects that ingested the saccharose solution with or without PTD-DRBD. Compared to these conditions, the administration of naked dsRNA decreased *Ag-ChSII* gene expression by approximately 30%, while its association with PTD-DRBD amplified the silencing effect, reducing its expression by ~80%. However, we observed that insect mortality was not significantly different between the different treatments over a period of 10 days (data not shown). This result indicates that PTD-DRBD complexed with dsRNA promotes *Ag-ChSII* gene silencing in *A. grandis*, although an enhancing effect on insect toxicity was not visible under our experimental conditions.

## Discussion

**Figure 7:**
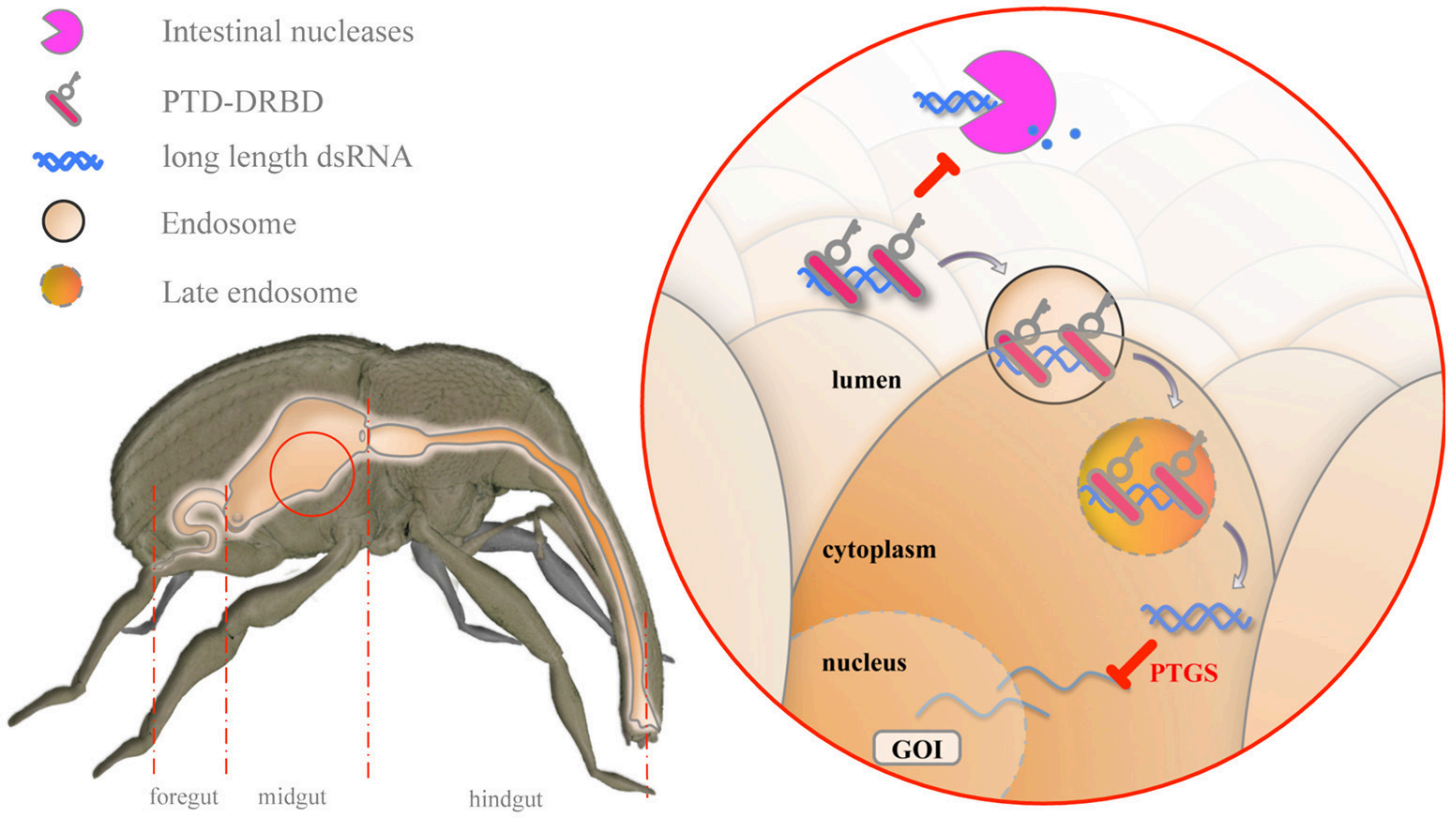
**Model for the mechanism of oral delivery of dsRNA combined with PTD-DRBD**. In the midgut, PTD-DRBD (red bar with key) covers the naked dsRNA (blue helix) and limits its degradation (blue dots) by gut-secreted nucleases (pink pacman). PTD stimulates endocytosis at the epithelial cell membrane, forming endosomes (lined circled RNP) and allowing endocytosis of the RNP. Endosomal maturation leads to endosomal acidification, which activates the fusogenic peptide. The release of dsRNA into the cytoplasm triggers the silencing of the gene of interest (GOI) in a sequence-specific manner.

Recent studies on CPPs have demonstrated the possibility of delivering molecules of interest to insect cells (Cermenati et al., 2011; Chen et al., 2012; Pan et al., 2014; Zhou et al., 2015). However, to the best of our knowledge, the use of CPPs to deliver dsRNA to crop insect pests has been restricted only to theoretical studies (Hughes et al., 2012). Herein, we show that an engineered RNP can be used to enhance dsRNA oral delivery in insects. Our data support a multistep mechanism that improves gene silencing in insect cells (Figure 7).

One main challenge for the use of RNAi-based plants against crop insect pests is stabilizing the dsRNA in the gut lumen, with secreted nucleases representing a limiting factor in this regard. We report that long dsRNAs in association with PTD-DRBD form RNPs that slow down the degradation of dsRNA by nucleases, probably by effectively covering and shielding the dsRNA. This molecular mechanism is especially consistent with certain viral strategies to counteract the host antiviral pathway in eukaryotes (Ding and Voinnet, 2007; Schnettler et al., 2009; Bivalkar-Mehla et al., 2011; Dickson and Wilusz, 2011; Hastie et al., 2012; Krug, 2014; Csorba et al., 2015). Similar to DRBD, some viral silencing repressors (VSR), such as FHV-B2 (flock house virus), TAV-2b (tomato aspermy virus) or H5N1-NS1 (influenza virus), electrostatically interact with the major and minor grooves of dsRNA along a restricted interface (Ryter and Schultz, 1998; Tian et al., 2004; Chao et al., 2005; Lingel et al., 2005; Chen et al., 2008; Cheng et al., 2009). In *ebolavirus*, VP35 binds to the backbone of dsRNA and caps its terminus via two different electrostatic interfaces (Kimberlin et al., 2010; Leung et al., 2010). In the H5N1-NS1 complex, a molecule of dsRNA is sequestered inside the tubular oligomeric structure of the viral RNP (Bornholdt and Prasad, 2008). These properties allow the avoidance of dsRNA recognition by host cells and, more specifically, by endonucleases, as reported *in vitro* based on Dicer-mediated cleavage assays (Chao et al., 2005; Fenner et al., 2006; Van Rij et al., 2006; Schnettler et al., 2010; Bronkhorst et al., 2014; Landeo-Ríos et al., 2016). Following the model of PTD-DRBD, certain VSRs, such as TBSV-P19 (tomato bushy stunt tombusvirus) and TAV-2b, have already been genetically modified to generate RNPs in animal cells (Park et al., 2014; Danielson et al., 2016). Optimizing the carrier protein to better fit the biological system could be envisaged, and the specific category of VSR represents a potential source of carrier protein that could be genetically engineered to improve RNP stability under these particular conditions.

It is worth mentioning that PTD-DRBD has been preliminarily engineered to deliver short hairpin RNAs (shRNAs) in animal cell culture, an environment that differs greatly from the gut lumen. Beyond the nucleases, the pH of the midgut homogenate is also an important parameter with a potential impact on RNP stability. In our study, we report that PTD-DRBD slows down dsRNA degradation at pH as low as 5.5 meaning that the chimeric protein still binds efficiently dsRNA in our experimental conditions. Similarly while the optimal pH of the VSR P19 to bind siRNA ranged from 6.2 to 7.6, its activity is still significant at pHs more acidic (Koukiekolo et al., 2007). Others DRBD could be engineered from proteins that showed an optimal dsRNA binding activity in acidic pHs (Fukuda et al., 2008). This being so, it should be considered that high stability of the RNPs could prevent the loading of dsRNA into the insect silencing machinery. As discussed by Danielson et al. (2016) in the case of chimeric Tat-P19 RNPs, its stability is context-dependent. The well-described P19 protein prevents the loading of siRNA in the Argonaute complex during viral infection but cannot inhibit its activation (Lakatos et al., 2004), perhaps because the complex subsequently processes dsRNA into single-stranded RNA (ssRNA). It seems that the efficiency of this VSR relies on its ability to sequester dsRNA and not to take it from the Argonaute complex. In addition to the high affinity of P19 for dsRNA, its high production inside cells supported by viral machinery might be another important parameter. In the context of siRNA delivery, the DRBD is supplied exogenously. In this case, it is possible that its cellular content is not enough to compete with the insect silencing machinery. Another factor interfering with RNP stability could be the maturation of vesicles into late endosomes during the endocytosis pathway, at which point the pH becomes highly acidic (El-Sayed and Harashima, 2013). This could contribute to the release of dsRNA inside the cytoplasm. To provoke RNP instability within the insect cell, other mechanisms of inactivation could be imagined using, for example, post-translational modifications of the carrier protein within the target cell that affect its affinity for dsRNA.

Another remarkable finding in this study was the short timescale necessary for *A. grandis* gut cells to internalize PTD. Similar observations have been reported in different animal cell models treated with cationic CPPs (Ziegler et al., 2005; Tünnemann et al., 2006; Rinne et al., 2007; Kosuge et al., 2008; Tanaka et al., 2012; Liu et al., 2013). While the molecular mechanism enabling the cellular entry of PTD in *A. grandis* insect cells is not identified, our data support the concept that PTD stimulates an endocytosis pathway. This type of molecular mechanism has been well described in other biological systems with different Tat-derived peptides, including PTD (Nakase et al., 2004; Wadia et al., 2004; Kaplan et al., 2005; Khalil et al., 2006; Tanaka et al., 2012). Specifically, it is tempting to speculate that PTD stimulates the macropinocytosis pathway in *A. grandis* and Sf21 cells. Endocytic pathways generally produce vesicles with a diameter below 0.2 μm, and macropinosomes can reach a diameter of 5–10 μm (Lim and Gleeson, 2011; El-Sayed and Harashima, 2013). In our study, the complex PTD-DRBD:dsRNA was observed in *A. grandis* cells enclosed in large vesicular bodies with diameters ranging from 600 nm to 2 μm. The large plasma membrane extensions observed in *A. grandis* and Sf21 cells also support this hypothesis.

The use of PTD and other CPPs illustrates the possibility of delivering dsRNA via a molecular mechanism distinct from the SID-1 dsRNA transporter pathways, which are thought to be a limiting step for dsRNA delivery in insect cells (Bellés, 2010; Katoch et al., 2013). Indeed, SID-1 selectively binds to dsRNA in a length-dependent manner. This selectivity is implicated in the poor binding of shRNA to SID-1 (Li et al., 2015) and its internalization (Bolognesi et al., 2012). However, compared to long-length dsRNAs, shRNAs provide better specificity in the silencing of a target gene and decrease the risk of off-target effects (Qiu et al., 2005; Kulkarni et al., 2006; Moffat et al., 2007). Beyond the ability of PTD-DRBD to improve the effectiveness of the RNAi technology, the RNPs also have the potential to improve its specificity in insect pests. Whether PTD-DRBD combined with shRNA provides efficient gene silencing in insect pests, however, remains to be established.

The CPPs represent a large peptide family with different biochemical characteristics (Laufer et al., 2012; Milletti, 2012; El-Sayed and Harashima, 2013). The choice of CPP is also probably significant in optimizing RNP stability. A previous EMSA study on PTD-DRBD with short dsRNA reported the interference of the positive charges of PTD in RNP formation (Geoghegan et al., 2012). Our analysis also points in that direction. In this configuration, PTD might decrease RNP stability and enhances its susceptibility to nuclease activity. The binding of PTD to dsRNA probably results in the production of a poorly organized RNP, causing its aggregation in a concentration-dependent manner. However we observed that the time of incubation of PTD-DRBD with dsRNA is a relevant parameter to ensure highest stability of the complex. This parameter was particularly determinant during our dsRNA cleavage assay mediated by nuclease. Alternatives to PTD for cellular internalization strategies could be envisaged. Designing a CPP specific to a crop insect pest is an attractive alternative as well.

Finally, the most important finding of this study was that the oral administration of RNP significantly improves gene silencing in *A. grandis*. However, we did not observe a link between the enhanced gene silencing effect of PTD-DRBD and the induction of toxicity in the *A. grandis* population after treatment. On the other hand, we did observe that the microinjection of *Ag-ChSII* dsRNA into the body cavity of adult insects causes high mortality in an *A. grandis* population (Lima et al., unpublished). It is possible that the silencing of *Ag-ChSII* was not adequate enough to suppress its function below a lethal level in the insect. Our assay involved a single oral administration, while continuous dsRNA ingestion, i.e., meal after meal, might result in a stronger induction of gene silencing (Jin et al., 2015; Zhang et al., 2015). Generating GM plants producing these engineered RNPs would allow us to explore this possibility.

Beyond its ability to increase gene silencing in insects, the use of RNPs could be particularly interesting for managing of the level of dsRNA expression in plant tissue. Indeed, one difficulty in the design of RNAi strategies in plants is regulating the expression of a gene of interest spatially and temporally. In most studies, dsRNA expression is combined with the use of strong constitutive promoters to compensate for poor RNAi efficiency in crop insect pests (Huvenne and Smagghe, 2010; Burand and Hunter, 2013; Katoch et al., 2013). High dsRNA production in plants increases the risk of off-target effects, either in the plant or in other organisms, potentially reducing the specificity of the pesticide. Today, a large number of tissue-specific or inducible promoters characterized from different plant species are available (Dutt et al., 2014). However, finding a promoter that reconciles specificity and high expression level could be challenging. The use of RNPs can compensate for the lower dsRNA expression of specific promoters in plant tissues by increasing dsRNA stability and delivery in insect cells. In this way, this tool can extend the panel of choices of plant tissue-specific promoters to those showing low expression levels. Whether RNPs can be produced and self-assembled in plant cells has yet to be established. The affinity of DRBD for dsRNA suggests the potential for interference of the protein with the plant gene silencing machinery, similar to VSRs (Ding and Voinnet, 2007). Interestingly, certain strategies have the advantages of sheltering dsRNA in chloroplasts to preserve it from the RNAi machinery in the cytoplasm (Jin et al., 2015; Zhang et al., 2015; Bally et al., 2016). Sheltering PTD-DRBD in chloroplasts would be an attractive way to allow RNP assembly without interfering with plant gene silencing.

## Concluding remarks

In this study, we provide a proof of concept showing that an engineered RNP can enhance the *in vitro* oral delivery of dsRNA in insects. Future investigations on the design of new fusion proteins adapted to the insect gut environment as well as the production of RNPs in plants represent attractive contributions to the further improvement of the oral delivery of dsRNA in crop insect pests.

## Materials and methods

### Rearing of *A. grandis*

Insects were reared from a colony growing at Embrapa Genetic Resource and Biotechnology (Brasilia, Brazil) under constant temperature (26 ± 2°C), relative humidity (70 ± 10%) and light (12-h photoperiod) conditions. They were fed daily with an artificial diet according to Monnerat et al. (2000).

### *A. grandis* midgut dissection and cell preparation

Adult *A. grandis* insects were immobilized on ice for 30 min. Each insect was then positioned in a sterile solution of phosphate buffer at pH 5.5 (10 mM Na_2_HPO_4_/KH_2_PO_4_, 2.7 mM KCl, and 137 mM NaCl), and dissection was performed under a stereomicroscope (Nikon SMZ1000). The elytra and wings were pulled off with forceps. The head was slowly detached from the thorax while taking care to not cut the esophagus. The head was then pulled until the entire digestive tract was removed from the body cavity (Figure S3). The midgut was separated from the foregut and the hindgut. The tissue was cut along the longitudinal axis and gently rinsed to eliminate the midgut homogenate. The midgut was transferred to an Eppendorf tube containing phosphate buffer at pH 5.5 and was immediately subjected to experimentation. Isolated midgut cells were obtained by leaving the midgut on a plate under agitation (100 rpm) in phosphate buffer at pH 5.5 at room temperature. Loosely attached cells were collected after 1 h of incubation and subjected to experimentation. The midgut homogenate preparation consisted of the expulsion of the midgut content onto a glass slide by soft compression of the midgut with forceps. The midgut homogenate was left to dry and then solubilized in 150 mM NaCl to obtain 5 μL of gut extract per dissected gut. The extract was pelleted using a microcentrifuge (14,000 rpm, 4°C), and the supernatant was stored at −20°C until use.

### Molecular cloning

Template DNA plasmids (pUC57Kan) containing the coding sequences for the PTD-eGFP and PTD-DRBD proteins were purchased from Epoch Biolabs Inc (Texas, USA). The plasmid pUC57Kan-PTD-eGFP was digested with NcoI restriction enzyme (New England Biolabs) to generate pUC57Kan-eGFP. Constructs were cloned into the pDEST24 vector (Invitrogen) using the Gateway cloning technology (Invitrogen) by following the manufacturer's recommendations. Full-length PTD-eGFP and PTD-DRBD fragments were amplified by PCR using Taq Platinum Polymerase (Invitrogen) with Gateway primers FXGWAttB1-S (forward) and FXGWAttB1-AS (reverse), (Table S1). Each construct was inserted into the pDONR221 vector (Invitrogen) following a BP clonase reaction and into the pDEST24 plasmid following the LR reaction. All DNA was sequenced on the both strands. The full sequences of each construct are available in the supplementary material section.

### Protein expression and purification

*E. coli* BL21 (DE3) Codon Plus (RIL) cells (Novagen) were used to express PTD-DRBD, PTD-eGFP or eGFP in 600 mL of LB in 2-L baffle flasks (200 rpm, 37°C). When cell growth reached an OD_600_ of approximately 0.2–0.4, cells were acclimated at 23°C. At an OD_600_ of approximately 0.7–0.9, protein expression was induced with 0.4 mM IPTG for 16–18 h. Cells were then collected by centrifugation (10 min, 4,000 rpm, 4°C) and resuspended in buffer A (50 mM Tris-HCl, pH 7.6, 0.5 M NaCl) at a volume 30 times lower than the initial culture volume. Afterward, cells were flash frozen and kept at −80°C until use. The extraction consisted of three sonication steps on ice using a sonifier (Branson Digital) with a 1/8″ tapered microtip (amplitude 20%, time 30″, interval 20″). The protein extract was centrifuged (14,000 rpm, 4°C), and the supernatant was filtered through a 0.45-μM PVDF membrane (Millipore). All of the purification steps were performed by fast protein liquid chromatography (FPLC) (AKTA pure 25 L, GE Healthcare Life Sciences). Protein extract was loaded onto a HisTrap FF 1-mL column preequilibrated with buffer A and 5% buffer B (buffer A + 0.5 M imidazole). The column was washed with buffer A, and the recombinant protein was eluted following a linear gradient of buffer B [5–100% over 30 CV−(column volume)]. Recombinant proteins were directly loaded onto a HisTrap FF SP 1-mL column (GE Healthcare Life Sciences) preequilibrated with buffer A. The elution was performed using buffer C (buffer A + 2 M NaCl) following a non-linear gradient (40%, 3 CV; 50%, 3 CV; 100%, 3 CV). The last purification step consisted of a buffer exchange against PBS at pH 7.4 (10 mM Na_2_HPO_4_, 1.8 mM KH_2_PO_4_, 2.7 mM KCl, 137 mM NaCl) using a 5-mL desalting column (GE Healthcare Life Sciences). The recombinant protein eGFP was purified following the same methodology. However, in the ion exchange step, the elution gradient was linear and stopped at 50% buffer C over 30 CV. The recombinant proteins concentration was evaluated based on UV absorbance, and then the solution was aliquoted, flash frozen and kept at −80°C.

### Electrophoretic mobility shift assay

The dsRNAs were purchased from Genolution Pharmaceuticals Inc. The dsRNA sequences are available in the supplementary data section. The EMSA was performed according to the protocol described by Hellman and Fried (2007). The recombinant proteins PTD-DRBD, PTD-eGFP and eGFP, initially stored at −80°C, were left on ice for 1 h before assay. Next, the dsRNAs and recombinant proteins were diluted to the desired concentrations in a binding buffer (50 mM Tris-acetate, pH 7.4, 5 mM EDTA, 1 mM DTT, 0.1 mg.ml^−1^ BSA, 10% glycerol, 0.015% NP-40, and 0.2 M NaCl). The proteins and dsRNA molarities in each EMSA experiment are described in the supplementary data section (Tables S2, S3). The mixture was homogenized by pipetting and left on ice for 20 min. The entire reaction volume was mixed with 5% glycerol and loaded onto a 5% acrylamide:bis-acrylamide (75:1) gel in TAE 1X buffer. Electrophoresis was performed at a constant voltage of 3.5 V/cm for 15 min and then 7 V/cm for 30 min. The gel was stained with 2 μM ethidium bromide (Sigma) for 10 min before on-gel fluorescence detection (ChemiDoc MP imaging system, Bio-Rad).

### Nuclease-mediated cleavage of DSRNA

The molarities used for the formation of PTD-DRBD:dsRNA and the dilutions of benzonase are described in Table S4. Benzonase (25 KU, Novagen 71206-3) was diluted in PBS at pH 7.4 to obtain a dilution range between 25 and 0.65 U. The concentrations of PTD-DRBD and dsRNA were 3.5 μM and 0.4 μM, respectively. The RNPs were incubated with benzonase for 20 min at room temperature before electrophoresis on a 1% agarose gel prestained with ethidium bromide (2 μM). The reaction was stopped with 3x “stop-and-load” buffer (89 mM Tris, 89 mM borate, 5 mM EDTA, 10 mM DEPC and 16% glycerol). for the experiment performed in non-denaturing conditions, while 5% SDS was added to the buffer when denaturing conditions were required. For the *A. grandis* gut nuclease-mediated cleavage assay, total soluble proteins (TSP) were titrated at 1.5 μg.μl^−1^ by Bradford assay using Bovine Serum Albumin (BSA) as standard (Bradford, 1970). PTD-DRBD (4 μM) and dsRNA (2 μM) were incubated 20 min on ice and 30 min at room temperature. Since PTD-DRBD was stored in a PBS buffer at pH7.4, the volume of a triple buffer was empirically evaluated to obtain a pH around 5.5 after mixing with the PBS buffer (Table S5). The final concentrations of PTD-DRBD and dsRNA were 1.6 μM and 0.2 μM, respectively. The final concentration of TSP from the midgut homogenate was 0.8 μg.μl^−1^ after dilution in the reaction mix. Throughout the 20 min of the reaction at room temperature, samples of 20 μL were removed from the mixture, 3x stop-and-load buffer containing 5% SDS was added, and the resulting solutions were flash frozen in liquid nitrogen. Samples were analyzed by electrophoresis in a 1.2% agarose gel stained in 2 μM ethidium bromide.

### Confocal microscopy

Fluorescence detection was performed using a Leica SP8 confocal laser scanning system with 100X (HCX PL FLUOTAR 100x/NA 1.30 oil) and 20X (HCX PL APO CS 20x/NA 0.70 DRY) objectives. dsRNA was conjugated with Cy3 fluorescent dye using the Cy3 Mono-Reactive Dye pack (Amersham, GE Healthcare) according to the manufacturer's instructions. dsRNA-Cy3 was excited at 552 nm (OP SL 552) and detected within a 552 nm-595 nm bandpass (HyD 1 detector, Leica). The membrane marker FM4-64 was prepared in DMSO or water as a working staining solution with a concentration of 5 μg/ml. Samples were treated with 2% (v/v) of FM4-64 for 5 min at room temperature before observation. The probe was excited at 552 nm (OP SL 552) and detected within a 734–800 nm bandpass (PMT). The DAPI fluorescent nuclear counter stain (Thermo Fisher) was prepared as a working solution at a concentration of 300 nM according to the manufacturer's instructions. The samples were stained with DAPI for 5 min at room temperature before observation. The fluorescent probe was excited at 405 nm (diode 405 nm) and detected within 416–482 nm (PMT). PTD-eGFP and eGFP fluorescent proteins were excited at 488 nm (OP SL 488) and detected within 493–531 nm (HyD 1). For time-lapse experiments, *A. grandis* and Sf21 cells were treated with 0.14 μM PTD-eGFP. The effect of bleaching on PTD-eGFP was evaluated over 20 min and determined to be minor at the laser intensity used (0.5–1%). Pictures from the time-lapse experiments were imported into the open-source ImageJ/Fiji software (Schindelin et al., 2012) to generate the movies.

### Assessment of the chitin synthase II expression pattern in *A. grandis* by quantitative real-time PCR

Adult *A. grandis* insects were fed with a 4 μL droplet of 5% sucrose containing dsRNA (0.6 μM or 500 ng) and PTD-DRBD (6.5 μM). Total RNA extraction from the whole *A. grandis* insect (*n* = 6) was performed 2 days after oral administration using the TRIzol reagent (Invitrogen-Life Technologies) according to the manufacturer's instructions. The RNA concentration was measured using UV spectrophotometry (NanoDrop, Thermo Fisher Scientific), and the quality was assessed on a 1% agarose gel. The PCR reaction was performed on a 7,300 Real-Time PCR System (Applied Biosystems) using SYBR Green as the intercalating fluorophore and specific primers for *Ag-ChSII* and for *Ag-*β*-Actin* and β*-Tubulin* genes, which were used as reference genes (Firmino et al., 2013), (Table S4). Each reaction was performed with 2 μL of a 1:20 cDNA dilution, 0.2 μM of each nucleotide and SYBR™ Green 4X at a total volume of 10 μL. The RTq-PCR program consisted of 95°C for 10 min and 40 cycles of 95°C for 20 s, 55°C for 30 s and 72°C for 30 s. For amplification analysis, the Ct value and amplification efficiency for each nucleotide (ranging from 90 to 100%) were determined using Real-time PCR Miner software (Zhao and Fernald, 2005). Relative expression analysis based on the Ct values and using β-actin as a reference gene was performed via the qBasePlus 2.0 method reported by Pfaffl (Hellemans et al., 2007). All qPCR experiments were performed with two biological replicates, which comprise for each 6 individuals beetles, and three technical repetitions. Statistical analysis was performed using Tukey's test with a 0.05% significance level for comparison between treatments.

## Author contributions

Conceptualization and writing-original draft and editing: FG; Data acquisition: FG and RG; Methodology: FG, RG, and LM; Resources: EA, MS, and MG; Writing–review: FG, RG, EA, MS, and MG.

## Funding

This work was supported by CAPES—Ciênca sem fronteiras (Project number: AO42/2013).

### Conflict of interest statement

The authors declare that the research was conducted in the absence of any commercial or financial relationships that could be construed as a potential conflict of interest.

## Supplementary material

Figure S1 **EMSA of long length dsRNA with PTD-DRBD. (A)** With a 150 nts length dsRNA, the concentration of PTD-DRBD ranged from 25 to 225 nM with a constant dsRNA concentration at 70 nM. The dsRNA sequence differs from the 185-nts length dsRNA used previously. **(B)** A 150 nts length dsRNA at 70 nM has been incubated with PTD-DRBD at a concentration ranging from 0.1 to 0.45 μM with a constant dsRNA concentration at 70 nM.

Figure S2 **EMSA of long length dsRNA with PTD-DRBD, PTD-eGFP or eGFP performed with ehidium bromide**. The assay was performed with 0.4 μM of dsRNA and with different concentrations of recombinant proteins (recPrt). Samples were separated by electrophoresis on 1.2% agarose gel pre-stained with 2 μM of ethidium bromide. Notably, at identical concentrations (i.e., 2.1 μM), the EtBr signal emitted from PTD-eGFP partially complexed with dsRNA is higher than PTD-DRBD fully complexed with dsRNA. This observation suggests that dsRNA seems to be more accessible by EtBr when the molecule is complexed with PTD-eGFP.

Figure S3 **The adult *A. grandis* digestive tract**. The picture was taken under visible light by a stereomacroscope.

Figure S4 **Evaluation of the pH in the *A. grandis* midgut homogenate**. The midgut homogenate has been diluted 10 times in fresh MQ water before applying on color-fixed pH indicator strips. The strips were scanned simultaneously with the corresponding indicator pads from **(A)** MN Macherey-Nagel (Germany) and **(B)** Qualividros, (Brazil).

Table S1 **Table of primers used in this study**.

Table S2 **Reaction mix used for the EMSA experiments–Figure 2**.

Table S3 **Reaction mix used for the EMSA experiments–Figure S2**.

Table S4 **Reaction mix used for the dsRNA protection assay against nucleases–Figure 3A**.

Table S5 **Reaction mix used for the dsRNA protection assay against gut nucleases–Figure 3B**.

Movie 1 **Time-lapse imaging showing the internalization of PTD-eGFP in *A. grandis* gut cell**. The yellow arrow indicates the detection of PTD-eGFP in an endovesicle.

Movie 2 **Time-lapse imaging showing in sf21 cells large plasma membrane movement of PTD-eGFP and its localization in an endovesicle**. White arrow indicates large plasma membrane extensions in contact with PTD-eGFP. Yellow arrows indicate the detection of PTD-eGFP in an endovesicle.
