# Supplementary figures and images for: Investigating Engineered Ribonucleoprotein Particles to Improve Oral RNAi Delivery in Crop Insect Pests
Source: Front Physiol. 2017 Apr 28;8:256. doi: 10.3389/fphys.2017.00256 (PMC5408074; doi:10.3389/fphys.2017.00256)

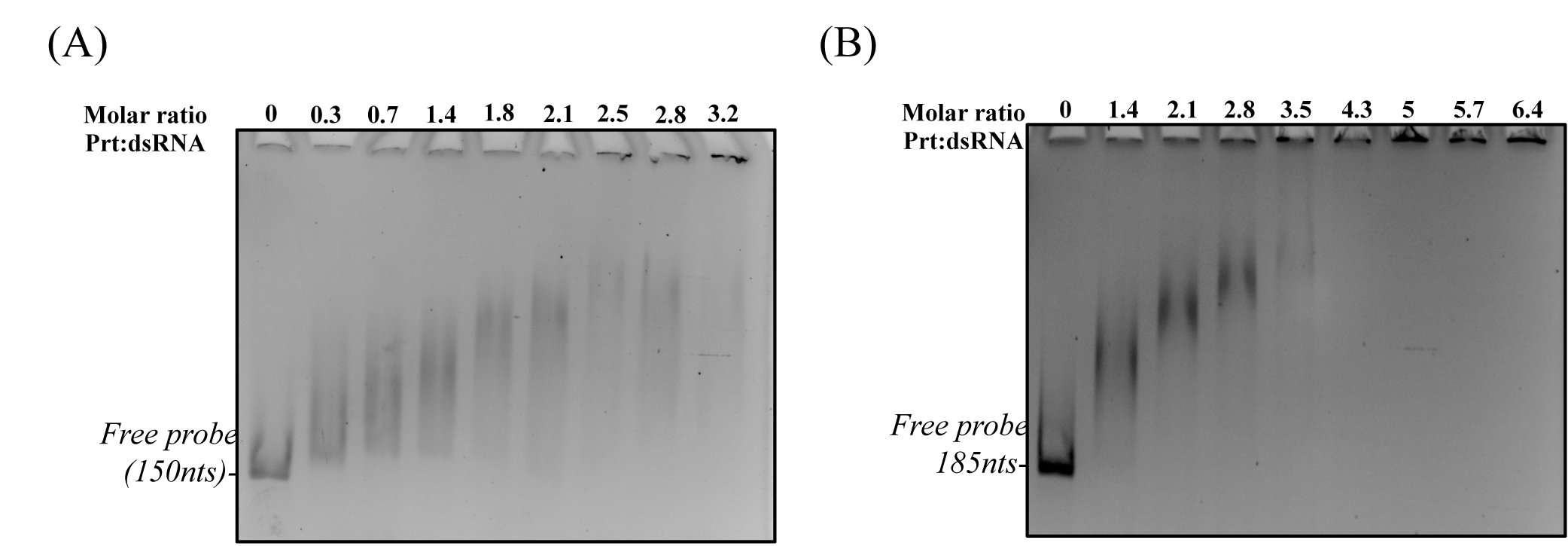

Supplement: Figure S1 — EMSA of long length dsRNA with PTD-DRBD. (A) With a 150 nts length dsRNA, the concentration of PTD-DRBD ranged from 25 to 225 nM with a constant dsRNA concentration at 70 nM. The dsRNA sequence differs from the 185-nts length dsRNA used previously. (B) A 150 nts length dsRNA at 70 nM has been incubated with PTD-DRBD at a concentration ranging from 0.1 to 0.45 μM with a constant dsRNA concentration at 70 nM. [file Image1.JPEG]

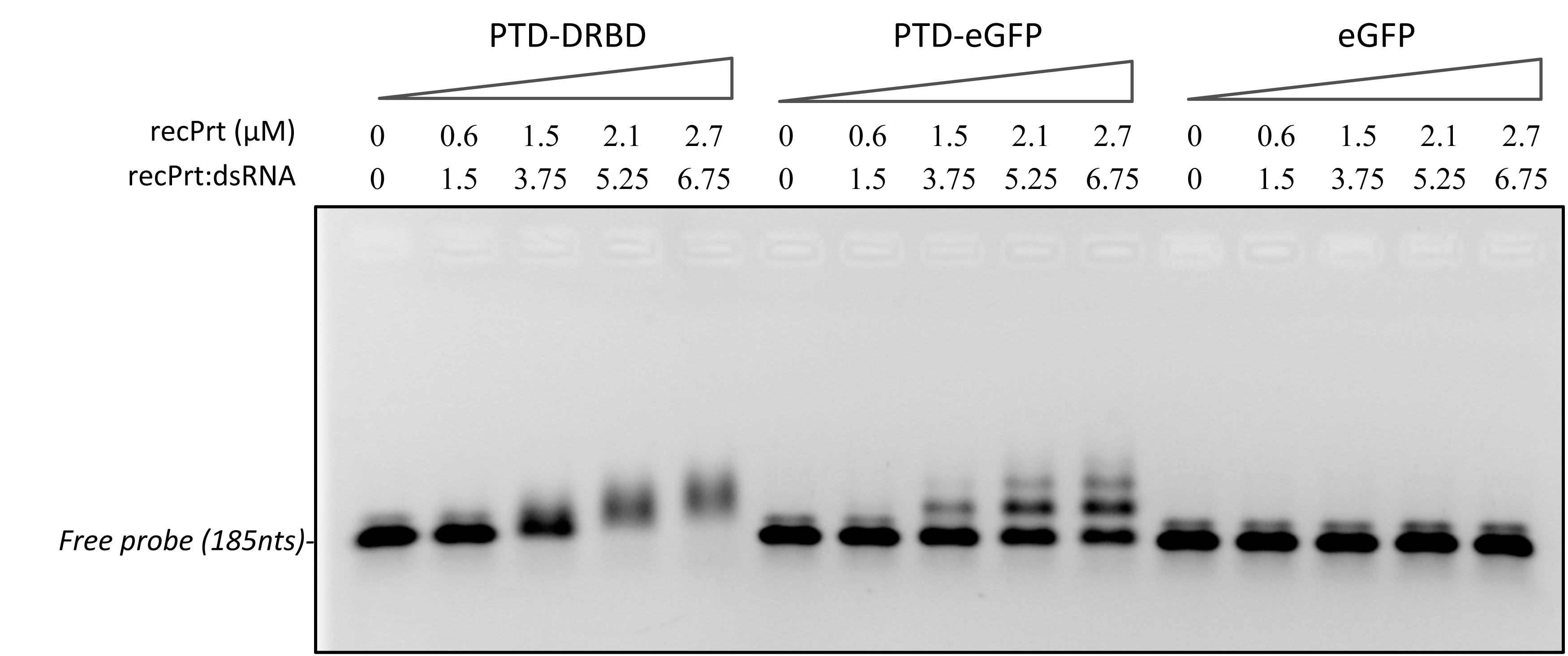

Supplement: Figure S2 — EMSA of long length dsRNA with PTD-DRBD, PTD-eGFP or eGFP performed with ehidium bromide. The assay was performed with 0.4 μM of dsRNA and with different concentrations of recombinant proteins (recPrt). Samples were separated by electrophoresis on 1.2% agarose gel pre-stained with 2 μM of ethidium bromide. Notably, at identical concentrations (i.e., 2.1 μM), the EtBr signal emitted from PTD-eGFP partially complexed with dsRNA is higher than PTD-DRBD fully complexed with dsRNA. This observation suggests that dsRNA seems to be more accessible by EtBr when the molecule is complexed with PTD-eGFP. [file Image2.JPEG]

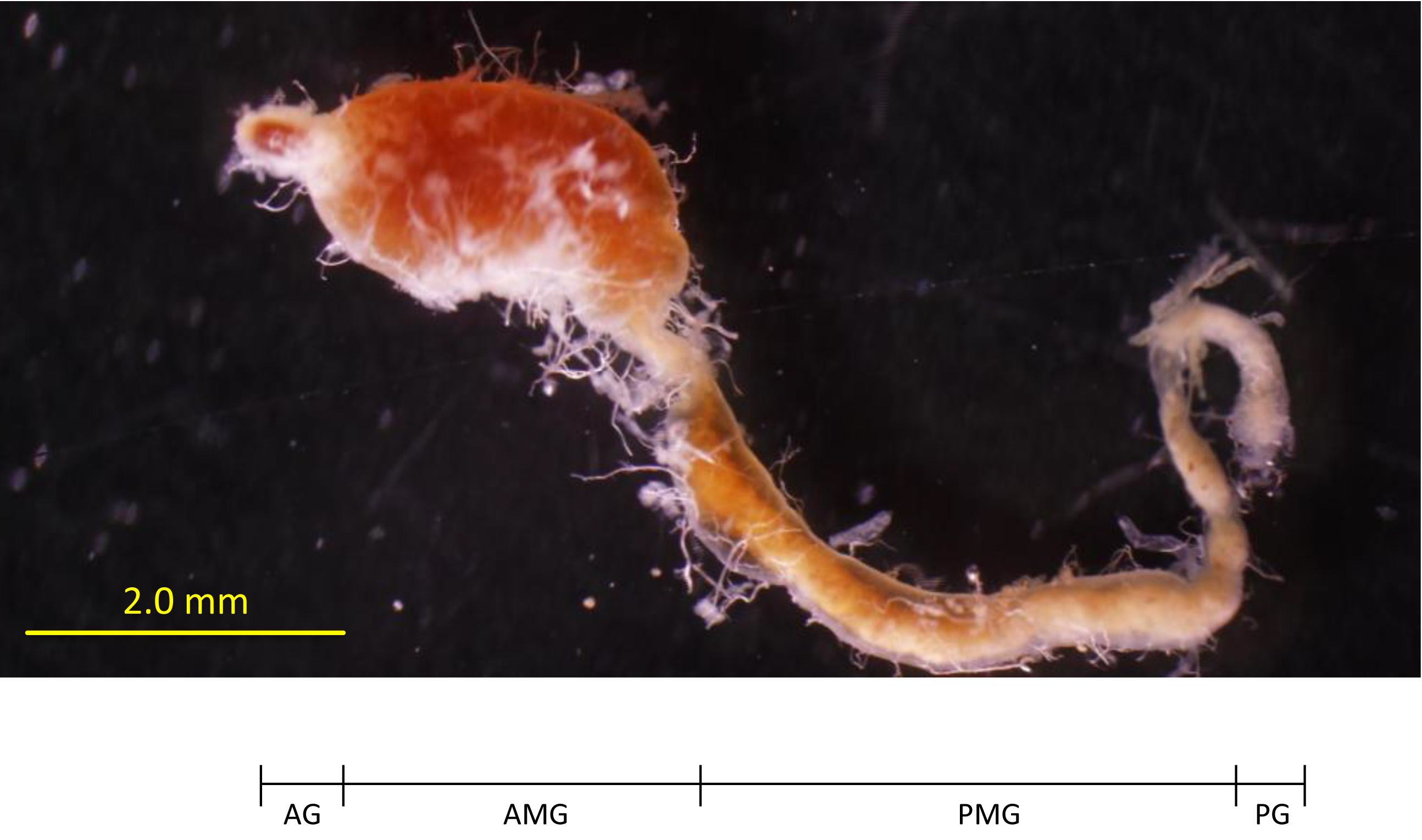

Supplement: Figure S3 — The adult A. grandis digestive tract. The picture was taken under visible light by a stereomacroscope. [file Image3.JPEG]

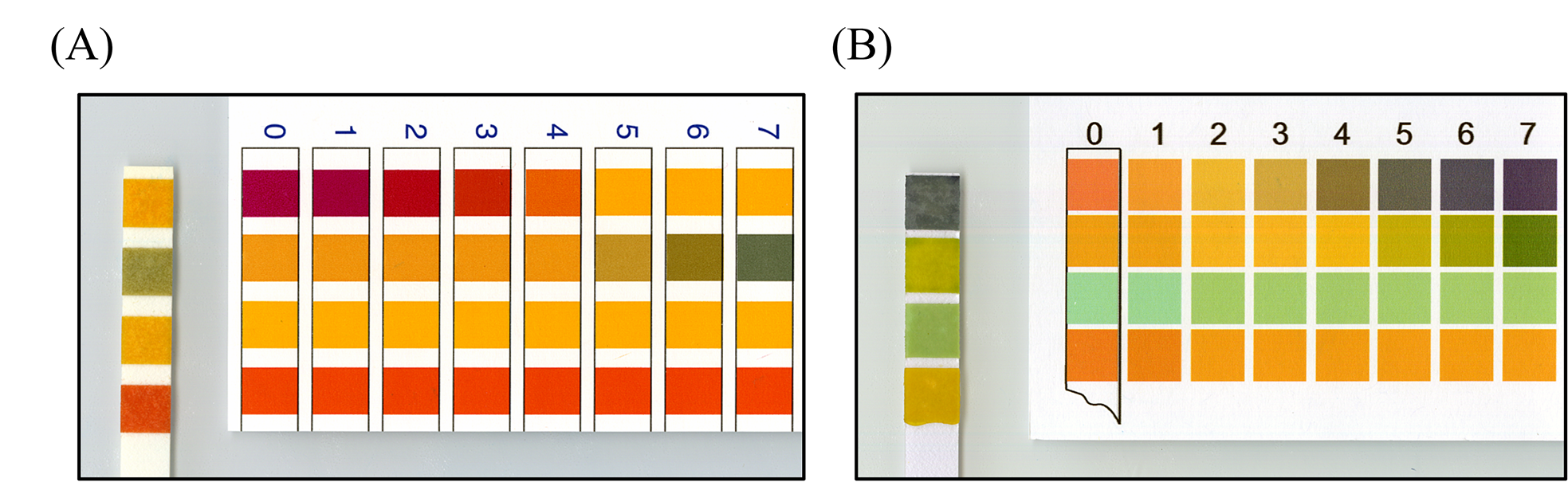

Supplement: Figure S4 — Evaluation of the pH in the A. grandis midgut homogenate. The midgut homogenate has been diluted 10 times in fresh MQ water before applying on color-fixed pH indicator strips. The strips were scanned simultaneously with the corresponding indicator pads from (A) MN Macherey-Nagel (Germany) and (B) Qualividros, (Brazil). [file Image4.TIF]
