# Supplementary material for: Investigating Engineered Ribonucleoprotein Particles to Improve Oral RNAi Delivery in Crop Insect Pests
Source: Front Physiol. 2017 Apr 28;8:256. doi: 10.3389/fphys.2017.00256 (PMC5408074; doi:10.3389/fphys.2017.00256)
Supplement: Table S1 — Table of primers used in this study. [file Presentation1.PDF]

Investigating engineered ribonucleoprotein particles to improve oral RNAi delivery in crop insect pests

Gillet et al.

SUPPLEMENTARY INFORMATIONS

LEGENDS SUPPLEMENTARY FIGURES

Figure S1: EMSA of long length dsRNA with PTD-DRBD. (A) With a 150nts length dsRNA, the concentration of PTD-DRBD ranged from 25nM to 225nM with a constant dsRNA concentration at 70nM. The dsRNA sequence differs from the 185-nts length dsRNA used previously. (**b**) A 150nts length dsRNA at 70nM has been incubated with PTD-DRBD at a concentration ranging from 0.1µM to 0.45µM with a constant dsRNA concentration at 70nM.

Figure S2: EMSA of long length dsRNA with PTD-DRBD, PTD-eGFP or eGFP performed with ehidium bromide. The assay was performed with 0.4µM of dsRNA and with different concentrations of recombinant proteins (recPrt). Samples were separated by electrophoresis on 1.2% agarose gel pre-stained with 2µM of ethidium bromide. Notably, at identical concentrations (i.e 2.1µM), the EtBr signal emitted from PTD-eGFP partially complexed with dsRNA is higher than PTD-DRBD fully complexed with dsRNA. This observation suggests that dsRNA seems to be more accessible by EtBr when the molecule is complexed with PTD-eGFP.

Figure S3: The adult *A. grandis* digestive tract. The picture was taken under visible light by a stereomacroscope.

Figure S4 : Evaluation of the pH in the *A.grandis* midgut homogenate. The midgut homogenate has been diluted 10 times in fresh MQ water before applying on color-fixed pH indicator strips. The strips were scanned simultaneously with the corresponding indicator pads from (A) MN Macherey-Nagel (Germany) and (B) Qualividros, (Brazil).

Figure S1

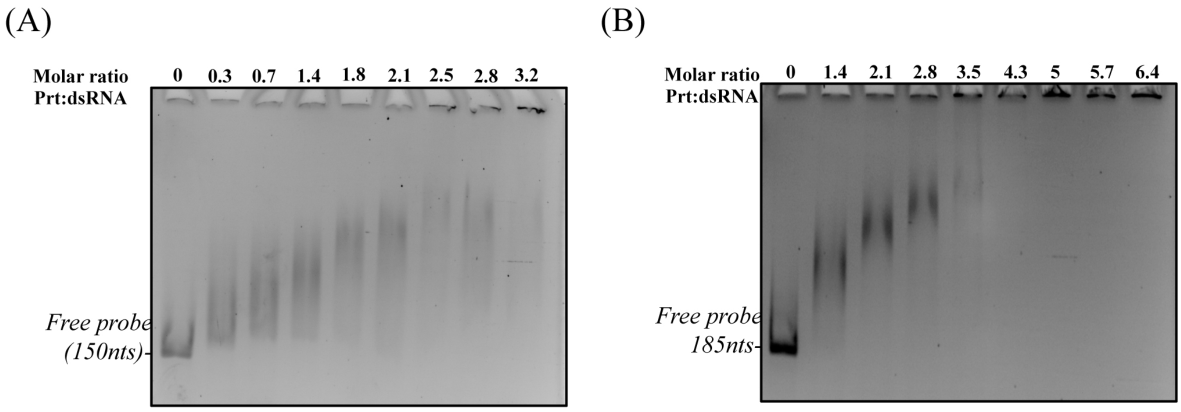
(A)
Molar ratio Prt:dsRNA
0 0.3 0.7 1.4 1.8 2.1 2.5 2.8 3.2
Free probe (150nts)
(B)
Molar ratio Prt:dsRNA
0 1.4 2.1 2.8 3.5 4.3 5 5.7 6.4
Free probe 185nts

Figure S2

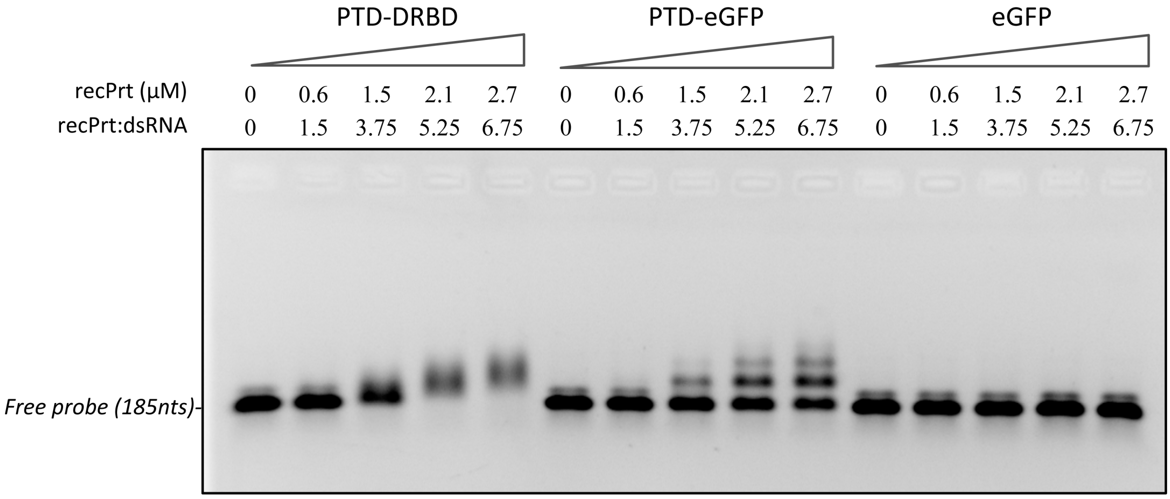
PTD-DRBD
PTD-eGFP
eGFP
recPrt (μM)
0 0.6 1.5 2.1 2.7 0 0.6 1.5 2.1 2.7 0 0.6 1.5 2.1 2.7
recPrt:dsRNA
0 1.5 3.75 5.25 6.75 0 1.5 3.75 5.25 6.75 0 1.5 3.75 5.25 6.75
Free probe (185nts)

Figure S3

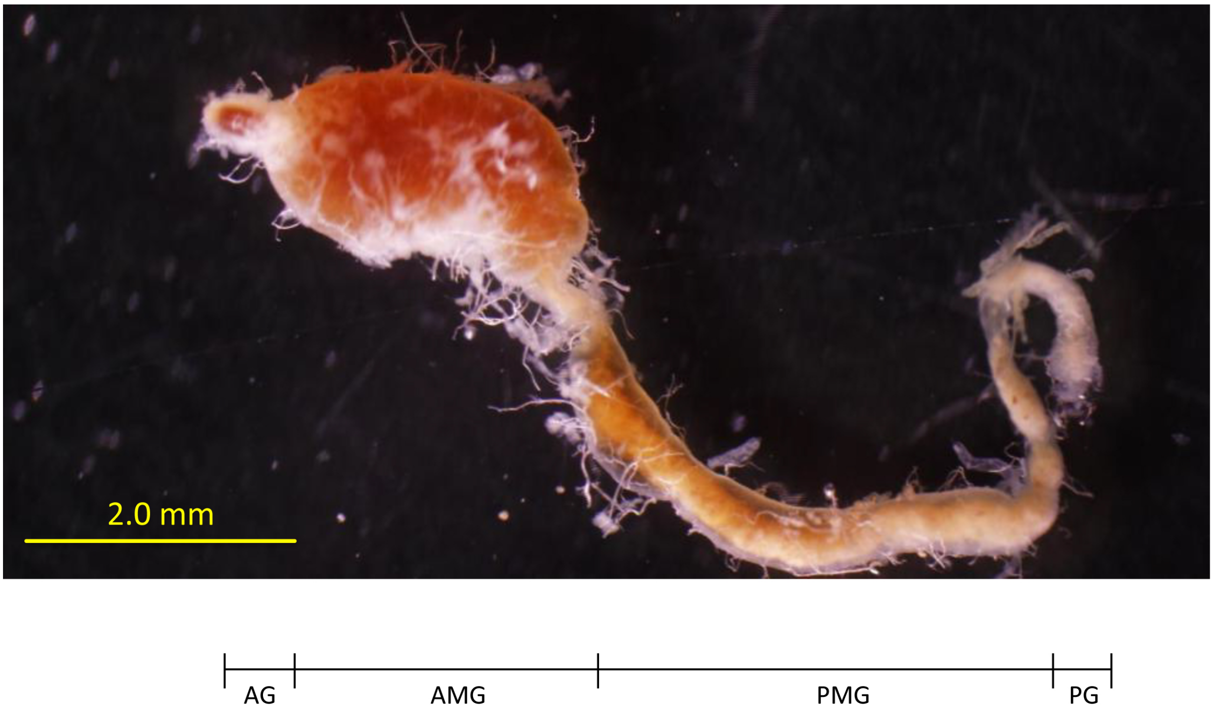

Figure S4

(A) (B)

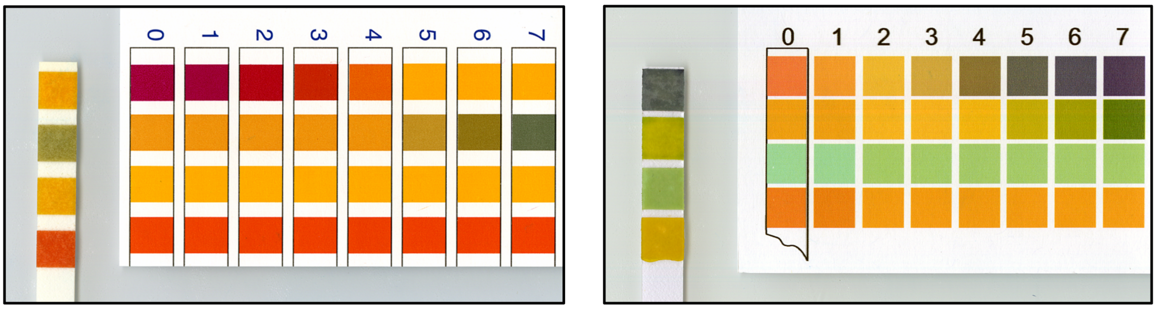

Molecular cloning

>pUC57Kan-eGFP

tcgcgcgtttcggtgatgacggtgaaaacctctgacacatgcagctcccggagacggtcacagcttgtctgtaagcggatgccgggagcagacaagcccgtcagggcgcgtcagcgggtgttggc
gggtgtcggggctggcttaactatgcggcatcagagcagattgtactgagagtgcaccatatgcggtgtgaaataccgcacagatgcgtaaggagaaaataccgcatcaggcgccattcgccatt
caggctgcgcaactgttgggaagggcgatcggtgcgggcctcttcgctattacgccagctggcgaaagggggatgtgctgcaaggcgattaagttgggtaacgccagggttttcccagtcacgac
gttgtaaaacgacggccagtgaattcgagctcggtacctcgcgaatgcatctagatatcggatcccggggacaagtttgtacaaaaaagcaggcttcgaaggagatagaaccatgggggtgagca
agggcgaggagctgttcaccggggtggtgcccatcctggtcgagctggacggcgacgtaaacggccacaagttcagcgtgtccggcgagggcgagggcgatgccacctacggcaagctgaccctg
aagttcatctgcaccaccggcaagctgcccgtgccctggcccaccctcgtgaccaccctgacctacggcgtgcagtgcttcagccgctaccccgaccacatgaagcagcacgacttcttcaagtc
cgccatgcccgaaggctacgtccaggagcgcaccatcttcttcaaggacgacggcaactacaagacccgcgccgaggtgaagttcgagggcgacaccctggtgaaccgcatcgagctgaagggca
tcgacttcaaggaggacggcaacatcctggggcacaagctggagtacaactacaacagccacaacgtctatatcatggccgacaagcagaagaacggcatcaaggtgaacttcaagatccgccac
aacatcgaggacggcagcgtgcagctcgccgaccactaccagcagaacacccccatcggcgacggccccgtgctgctgcccgacaaccactacctgagcacccagtccgccctgagcaaagaccc
caacgagaagcgcgatcacatggtcctgctggagttcgtgaccgccgccgggatcactctcggcatggacgagctgtacaaggcacaccaccaccaccaccactgacccggggacccagctttct
tgtacaaagtggtccccgggcccgtcgactgcagaggcctgcatgcaagcttggcgtaatcatggtcatagctgtttcctgtgtgaaattgttatccgctcacaattccacacaacatacgagcc
ggaagcataaagtgtaaagcctggggtgcctaatgagtgagctaactcacattaattgcgttgcgctcactgcccgctttccagtcgggaaacctgtcgtgccagctgcattaatgaatcggcca
acgcgcggggagaggcggtttgcgtattgggcgcggccgccgcttcctcgctcactgactcgctgcgctcggtcgttcggctgcggcgagcggtatcagctcactcaaaggcggtaatacggtta
tccacagaatcaggggataacgcaggaaagaacatgtgagcaaaaggccagcaaaaggccaggaaccgtaaaaaggccgcgttgctggcgtttttccataggctccgcccccctgacgagcatca
caaaaatcgacgctcaagtcagaggtggcgaaacccgacaggactataaagataccaggcgtttccccctggaagctccctcgtgcgctctcctgttccgaccctgccgcttaccggatacctgt
ccgcctttctcccttcgggaagcgtggcgctttctcatagctcacgctgtaggtatctcagttcggtgtaggtcgttcgctccaagctgggctgtgtgcacgaaccccccgttcagcccgaccgc
tgcgccttatccggtaactatcgtcttgagtccaacccggtaagacacgacttatcgccactggcagcagccactggtaacaggattagcagagcgaggtatgtaggcggtgctacagagttctt
gaagtggtggcctaactacggctacactagaagaacagtatttggtatctgcgctctgctgaagccagttaccttcggaaaaagagttggtagctcttgatccggcaaacaaaccaccgctggta
gcggtggtttttttgtttgcaagcagcagattacgcgcagaaaaaaaggatctcaagaagatcctttgatcttttctacggggtctgacgctcagtggaacgaaaactcacgttaagggattttg
gtcatgagattatcaaaaaggatcttcacctagatccttttaaattaaaaatgaagttttaaatcaatctaaagtatatatgagtaaacttggtctgacagttagaaaaactcatcgagcatcaa
atgaaactgcaatttattcatatcaggattatcaataccatatttttgaaaaagccgtttctgtaatgaaggagaaaactcaccgaggcagttccataggatggcaagatcctggtatcggtctg
cgattccgactcgtccaacatcaatacaacctattaatttcccctcgtcaaaaataaggttatcaagtgagaaatcaccatgagtgacgactgaatccggtgagaatggcaaaagtttatgcatt
tctttccagacttgttcaacaggccagccattacgctcgtcatcaaaatcactcgcatcaaccaaaccgttattcattcgtgattgcgcctgagcgagacgaaatacgcgatcgctgttaaaagg
acaattacaaacaggaatcgaatgcaaccggcgcaggaacactgccagcgcatcaacaatattttcacctgaatcaggatattcttctaatacctggaatgctgttttcccagggatcgcagtgg
tgagtaaccatgcatcatcaggagtacggataaaatgcttgatggtcggaagaggcataaattccgtcagccagtttagtctgaccatctcatctgtaacatcattggcaacgctacctttgcca
tgtttcagaaacaactctggcgcatcgggcttcccatacaatcgatagattgtcgcacctgattgcccgacattatcgcgagcccatttatacccatataaatcagcatccatgttggaatttaa
tcgcggcctagagcaagacgtttcccgttgaatatggctcatactcttcctttttcaatattattgaagcatttatcagggttattgtctcatgagcggatacatatttgaatgtatttagaaaa
ataaacaaataggggttccgcgcacatttccccgaaaagtgccacctgacgtctaagaaaccattattatcatgacattaacctataaaaataggcgtatcacgaggccctttcgtc

Colour code: eGFP, AttB1 and 2 boxes for gateway reaction

>pUCKan-PTD-DRBD

tcgcgcgtttcggtgatgacggtgaaaacctctgacacatgcagctcccggagacggtcacagcttgtctgtaagcggatgccgggagcagacaagcccgtcagggcgcgtcagcgggtgttggc
gggtgtcggggctggcttaactatgcggcatcagagcagattgtactgagagtgcaccatatgcggtgtgaaataccgcacagatgcgtaaggagaaaataccgcatcaggcgccattcgccatt
caggctgcgcaactgttgggaagggcgatcggtgcgggcctcttcgctattacgccagctggcgaaagggggatgtgctgcaaggcgattaagttgggtaacgccagggttttcccagtcacgac
gttgtaaaacgacggccagtgaattcgagctcggtacctcgcgaatgcatctagatatcggatcccggggacaagtttgtacaaaaaagcaggcttcgaaggagatagaaccatgggcaggaaga
agcggagacagcgacgaagaggccatagcggccgtaaaaagcgtcgtcagcgtcgtcgtggccatatttatccgtacgatgtgccggactatgcgggagatccgggtcgtaagaagcgccgtcag
cgccgtcgcggggacccggctggtgatctttcaccgggtgcggccgctttcttcatggaggaacttaatacataccgtcagaagcagggagtagtacttaaatatcaagaactgcctaattcagg
acctccacatgataggaggtttacatttcaagttataatagatggaagagaatttccagaaggtgaaggtagatcaaagaaggaagcaaaaaatgccgcagccaaattagctgttgagatactta
ataaggaaaagaaggcagccgcactcgagcaccaccaccaccaccactgagaattctagtcgactaggatccgacccagctttcttgtacaaagtggtccccgggcccgtcgactgcagaggcct
gcatgcaagcttggcgtaatcatggtcatagctgtttcctgtgtgaaattgttatccgctcacaattccacacaacatacgagccggaagcataaagtgtaaagcctggggtgcctaatgagtga
gctaactcacattaattgcgttgcgctcactgcccgctttccagtcgggaaacctgtcgtgccagctgcattaatgaatcggccaacgcgcggggagaggcggtttgcgtattgggcgcggccgc
cgcttcctcgctcactgactcgctgcgctcggtcgttcggctgcggcgagcggtatcagctcactcaaaggcggtaatacggttatccacagaatcaggggataacgcaggaaagaacatgtgag
caaaaggccagcaaaaggccaggaaccgtaaaaaggccgcgttgctggcgtttttccataggctccgcccccctgacgagcatcacaaaaatcgacgctcaagtcagaggtggcgaaacccgaca

ggactataaagataccaggcgtttccccctggaagctccctcgtgcgctctcctgttccgaccctgccgcttaccggatacctgtccgcctttctcccttcgggaagcgtggcgctttctcatag

ctcacgctgtaggtatctcagttcggtgtaggtcgttcgctccaagctgggctgtgtgcacgaaccccccgttcagcccgaccgctgcgccttatccggtaactatcgtcttgagtccaacccgg

taagacacgacttatcgccactggcagcagccactggtaacaggattagcagagcgaggtatgtaggcggtgctacagagttcttgaagtggtggcctaactacggctacactagaagaacagta

tttggtatctgcgctctgctgaagccagttaccttcggaaaaagagttggtagctcttgatccggcaaacaaaccaccgctggtagcggtggtttttttgtttgcaagcagcagattacgcgcag

aaaaaaaggatctcaagaagatcctttgatcttttctacggggtctgacgctcagtggaacgaaaactcacgttaagggattttggtcatgagattatcaaaaaggatcttcacctagatcctttt

taaattaaaaatgaagttttaaatcaatctaaagtatatatgagtaaacttggtctgacagttagaaaaactcatcgagcatcaaatgaaactgcaatttattcatatcaggattatcaatacca

tatttttgaaaaagccgtttctgtaatgaaggagaaaactcaccgaggcagttccataggatggcaagatcctggtatcggtctgcgattccgactcgtccaacatcaatacaacctattaattt

cccctcgtcaaaaataaggttatcaagtgagaaatcaccatgagtgacgactgaatccggtgagaatggcaaaagtttatgcatttctttccagacttgttcaacaggccagccattacgctcgt

catcaaaatcactcgcatcaaccaaaccgttattcattcgtgattgcgcctgagcgagacgaaatacgcgatcgctgttaaaaggacaattacaaacaggaatcgaatgcaaccggcgcaggaac

actgccagcgcatcaacaatattttcacctgaatcaggatattcttctaatacctggaatgctgttttcccagggatcgcagtggtgagtaaccatgcatcatcaggagtacggataaaatgctt

gatggtcggaagaggcataaattccgtcagccagtttagtctgaccatctcatctgtaacatcattggcaacgctacctttgccatgtttcagaaacaactctggcgcatcgggcttcccataca

atcgatagattgtcgcacctgattgcccgacattatcgcgagcccatttatacccatataaatcagcatccatgttggaatttaatcgcggcctagagcaagacgtttcccgttgaatatggctc

atactcttcctttttcaatattattgaagcatttatcagggttattgtctcatgagcggatacatatttgaatgtatttagaaaaataaacaaataggggttccgcgcacatttccccgaaaagt

gccacctgacgtctaagaaaccattattatcatgacattaacctataaaaataggcgtatcacgaggccctttcgtc

Colour code: PTD-DRBD, AttB1 and 2 boxes for gateway reaction

>pUCKan-PTD-eGFP

tcgcgcgtttcggtgatgacggtgaaaacctctgacacatgcagctcccggagacggtcacagcttgtctgtaagcggatgccgggagcagacaagcccgtcagggcgcgtcagcgggtgttggc

gggtgtcggggctggcttaactatgcggcatcagagcagattgtactgagagtgcaccatatgcggtgtgaaataccgcacagatgcgtaaggagaaaataccgcatcaggcgccattcgccatt

caggctgcgcaactgttgggaagggcgatcggtgcgggcctcttcgctattacgccagctggcgaaagggggatgtgctgcaaggcgattaagttgggtaacgccagggttttcccagtcacgac

gttgtaaaacgacggccagtgaattcgagctcggtacctcgcgaatgcatctagatatcggatcccggggacaagtttgtacaaaaaagcaggcttcgaaggagatagaaccatgggcaggaaga

agcggagacagcgacgaagaggccatagcggccgtaaaaagcgtcgtcagcgtcgtcgtggccatatttatccgtacgatgtgccggactatgcgggagatccgggtcgtaagaagcgccgtcag

cgccgtcgcggggacccggctggtgatcttggagccatgggggtgagcaagggcgaggagctgttcaccggggtggtgcccatcctggtcgagctggacggcgacgtaaacggccacaagttcag

cgtgtccggcgagggcgagggcgatgccacctacggcaagctgaccctgaagttcatctgcaccaccggcaagctgcccgtgccctggcccaccctcgtgaccaccctgacctacggcgtgcagt

gcttcagccgctaccccgaccacatgaagcagcacgacttcttcaagtccgccatgcccgaaggctacgtccaggagcgcaccatcttcttcaaggacgacggcaactacaagacccgcgccgag

gtgaagttcgagggcgacaccctggtgaaccgcatcgagctgaagggcatcgacttcaaggaggacggcaacatcctggggcacaagctggagtacaactacaacagccacaacgtctatatcat

ggccgacaagcagaagaacggcatcaaggtgaacttcaagatccgccacaacatcgaggacggcagcgtgcagctcgccgaccactaccagcagaacacccccatcggcgacggccccgtgctgc

tgcccgacaaccactacctgagcacccagtccgccctgagcaaagaccccaacgagaagcgcgatcacatggtcctgctggagttcgtgaccgccgccgggatcactctcggcatggacgagctg

tacaaggcacaccaccaccaccaccactgacccggggacccagctttcttgtacaaagtggtccccgggcccgtcgactgcagaggcctgcatgcaagcttggcgtaatcatggtcatagctgtt

tcctgtgtgaaattgttatccgctcacaattccacacaacatacgagccggaagcataaagtgtaaagcctggggtgcctaatgagtgagctaactcacattaattgcgttgcgctcactgcccg

ctttccagtcgggaaacctgtcgtgccagctgcattaatgaatcggccaacgcgcggggagaggcggtttgcgtattgggcgcggccgccgcttcctcgctcactgactcgctgcgctcggtcgt

tcggctgcggcgagcggtatcagctcactcaaaggcggtaatacggttatccacagaatcaggggataacgcaggaaagaacatgtgagcaaaaggccagcaaaaggccaggaaccgtaaaaagg

ccgcgttgctggcgtttttccataggctccgcccccctgacgagcatcacaaaaatcgacgctcaagtcagaggtggcgaaacccgacaggactataaagataccaggcgtttccccctggaagc

tccctcgtgcgctctcctgttccgaccctgccgcttaccggatacctgtccgcctttctcccttcgggaagcgtggcgctttctcatagctcacgctgtaggtatctcagttcggtgtaggtcgt

tcgctccaagctgggctgtgtgcacgaaccccccgttcagcccgaccgctgcgccttatccggtaactatcgtcttgagtccaacccggtaagacacgacttatcgccactggcagcagccactg

gtaacaggattagcagagcgaggtatgtaggcggtgctacagagttcttgaagtggtggcctaactacggctacactagaagaacagtatttggtatctgcgctctgctgaagccagttaccttc

ggaaaaagagttggtagctcttgatccggcaaacaaaccaccgctggtagcggtggtttttttgtttgcaagcagcagattacgcgcagaaaaaaaggatctcaagaagatcctttgatcttttc

tacggggtctgacgctcagtggaacgaaaactcacgttaagggattttggtcatgagattatcaaaaaggatcttcacctagatccttttaaattaaaaatgaagttttaaatcaatctaaagta

tatatgagtaaacttggtctgacagttagaaaaactcatcgagcatcaaatgaaactgcaatttattcatatcaggattatcaataccatatttttgaaaaagccgtttctgtaatgaaggagaa

aactcaccgaggcagttccataggatggcaagatcctggtatcggtctgcgattccgactcgtccaacatcaatacaacctattaatttcccctcgtcaaaaataaggttatcaagtgagaaatc

accatgagtgacgactgaatccggtgagaatggcaaaagtttatgcatttctttccagacttgttcaacaggccagccattacgctcgtcatcaaaatcactcgcatcaaccaaaccgttattca

ttcgtgattgcgcctgagcgagacgaaatacgcgatcgctgttaaaaggacaattacaaacaggaatcgaatgcaaccggcgcaggaacactgccagcgcatcaacaatattttcacctgaatca

ggatattcttctaatacctggaatgctgttttcccagggatcgcagtggtgagtaaccatgcatcatcaggagtacggataaaatgcttgatggtcggaagaggcataaattccgtcagccagtt

tagtctgaccatctcatctgtaacatcattggcaacgctacctttgccatgtttcagaaacaactctggcgcatcgggcttcccatacaatcgatagattgtcgcacctgattgcccgacattat

cgcgagcccatttatacccatataaatcagcatccatgttggaatttaatcgcggcctagagcaagacgtttcccgttgaatatggctcatactcttcctttttcaatattattgaagcatttat

cagggttattgtctcatgagcggatacatatttgaatgtatttagaaaaataaacaaataggggttccgcgcacatttccccgaaaagtgccacctgacgtctaagaaaccattattatcatgac

attaacctataaaaataggcgtatcacgaggccctttcgtc

Colour code: PTD-eGFP, AttB1 and 2 boxes for gateway reaction

Sequence dsRNA only the sense part

Sequence DsRNA

>Agra_CHS2-3

AAGTAGACGCTCACGTATCCAGAAGGAAAACCGTGGCGGACTTGGCGAAAAACAAAGACAGAAAACGTGCAGTCATCAACGACTTGGATTCCGCCTTTTAAAGCCCGTATGGCAAAAAATACGTA

AAGGAGAAGACGCGTGGAGTGGCTTTACCTAGGAAAACTTTGGCAGCAATTCACAGAAGA

>dsRNA_ICL

ACCTCTAGTTGCTAATGTGTAATCAATATCAATAGATCCTCTCAATTTCAACAAATCCTCAACTTCATAATTTCGTTTAATTCCCTTGTAACGACCCTTTGGTGCAGTCTTTACCACTTGATAAA

AATTTTTGGCGATAGTCTGTGACAT

Table S1 – Table of primers used in this study

Primer	Sequence (5'to 3')
AgCHSII Fw	AAGGCATTAACGGTGACGAC
AgCHSII Rv	TCCAAGTCGTTGATGACTGC
B-actin Fw	GTAGCTCACGCCTCGGTACT
B-actin Rv	AGTGTTGGCCGAGGTATGAC
B-tubulin Fw	GGTTGCGACTGTTTACAAGG
B-tubulin Rv	GCACCACCGAGTAAGTGTTC
FXGWAttB1-S	GGGGACAAGTTTGTACAAAA
FXGWAttB1-AS	GGGGACCACTTTGTACAAG

Table S2 – Reaction mix used for the EMSA experiments – Figure 2

Ratio RecPrt:dsRNA	0	0.35	0.71	1.43	1.78	2.14	2.5	2.85	3.21
V(recPrt)	0µl	1µl	2µl	3µl	4µl	5µl	6µl	7µl	8µl
[recPrt]f	0µM	0.025µM	0.05µM	0.1µM	0.125µM	0.150µM	0.175µM	0.2µM	0.225µM
V(dsRNA)	0.5µL	0.5µL	0.5µL	0.5µL	0.5µL	0.5µL	0.5µL	0.5µL	0.5µL
[dsRNA]f	0.07µM	0.07µM	0.07µM	0.07µM	0.07µM	0.07µM	0.07µM	0.07µM	0.07µM
PBS pH 7.4	9.5µl	8.5µl	7.5µl	6.5µl	5.5µl	4.5µl	3.5µl	2.5µl	1.5µl

Table S3 – Reaction mix used for the EMSA experiments – Figure S2

Ratio RecPrt:dsRNA	0	1.5	3.75	5.25	6.75
V(recPrt) in µl	0	2	5	7	9
$[recPrt]_f$ in µM	0	0.6	1.5	2.1	2.7
V(dsRNA) in µl	0.5	0.5	0.5	0.5	0.5
$[dsRNA]_f$ in µM	0.4	0.4	0.4	0.4	0.4
TBE 0.5X in µl	9	7	5	2	0

Table S4 – Reaction mix used for the dsRNA protection assay against nucleases – Figure 3a

V(PTD-DRBD) in µl	4	4	4	4	4	4
[PTD-DRBD]$_f$ in µM	3.5	3.5	3.5	3.5	3.5	3.5
dsRNA in µM	0.5	0.5	0.5	0.5	0.5	0.5
	0.4	0.4	0.4	0.4	0.4	0.4
V(Benzonase) in µl	0.5	0.5	0.5	0.5	0.5	0.5
Units	0U	25U	12.5U	2.5U	1.25U	0.65U

Table S5 – Reaction mix used for the dsRNA protection assay against gut nucleases – Figure 3b

<table>
<tr><td rowspan="2">Mix 1</td><td>PTD-DRBD (4µM) in PBS pH7.4</td><td>48µl</td><td rowspan="2">Mix 2</td><td>gut extract (1.5$\mu g.\mu L^{-1}$ of TSP)</td><td>6µl</td></tr>
<tr><td>dsRNA (2µM) in PBS pH7.4</td><td>6µl</td><td>Triple buffer pH 4.3</td><td>66µl</td></tr>
<tr><td colspan="3">20min on ice ; 30min at room temperature</td><td colspan="3">50min on ice</td></tr>
<tr><td colspan="2">PTD-DRBD:dsRNA (Mix 1)</td><td colspan="4">54µl</td></tr>
<tr><td colspan="2">Gut in triple buffer (Mix 2)</td><td colspan="4">72µl</td></tr>
<tr><td colspan="6">20min at room temperature</td></tr>
</table>
